# Interleukin-36γ and IL-36 receptor signaling mediate impaired host immunity and lung injury in cytotoxic *Pseudomonas aeruginosa* pulmonary infection: Role of prostaglandin E2

**DOI:** 10.1371/journal.ppat.1006737

**Published:** 2017-11-22

**Authors:** Tetsuji Aoyagi, Michael W. Newstead, Xianying Zeng, Yuta Nanjo, Marc Peters-Golden, Mitsuo Kaku, Theodore J. Standiford

**Affiliations:** 1 Division of Pulmonary and Critical Care Medicine, Department of Internal Medicine, University of Michigan, Ann Arbor, Michigan, United States of America; 2 Department of Infection Control and Laboratory Diagnostics, Internal Medicine, Tohoku University Graduate School of Medicine, Sendai, Japan; 3 Department of Microbiology and Infectious Diseases, Toho University School of Medicine, Tokyo, Japan; University of São Paulo FMRP/USP, BRAZIL

## Abstract

*Pseudomonas aeruginosa* is a Gram-negative pathogen that can lead to severe infection associated with lung injury and high mortality. The interleukin (IL)-36 cytokines (IL-36α, IL-36β and IL-36γ) are newly described IL-1 like family cytokines that promote inflammatory response via binding to the IL-36 receptor (IL-36R). Here we investigated the functional role of IL-36 cytokines in the modulating of innate immune response against *P*. *aeruginosa* pulmonary infection. The intratracheal administration of flagellated cytotoxic *P*. *aeruginosa* (ATCC 19660) upregulated IL-36α and IL-36γ, but not IL-36β, in the lungs. IL-36α and IL-36γ were expressed in pulmonary macrophages (PMs) and alveolar epithelial cells in response to *P*. *aeruginosa in vitro*. Mortality after bacterial challenge in IL-36 receptor deficient (IL-36R^-/-^) mice and IL-36γ deficient (IL-36γ^-/-^) mice, but not IL-36α deficient mice, was significantly lower than that of wild type mice. Decreased mortality in IL-36R^-/-^ mice and IL-36γ^-/-^ mice was associated with reduction in bacterial burden in the alveolar space, bacterial dissemination, production of inflammatory cytokines and lung injury, without changes in lung leukocyte influx. Interestingly, IL-36γ enhanced the production of prostaglandin E2 (PGE2) during *P*. *aeruginosa* infection *in vivo* and *in vitro*. Treatment of PMs with recombinant IL-36γ resulted in impaired bacterial killing via PGE2 and its receptor; EP2. *P*. *aeruginosa* infected EP2 deficient mice or WT mice treated with a COX-2-specific inhibitor showed decreased bacterial burden and dissemination, but no change in lung injury. Finally, we observed an increase in IL-36γ, but not IL-36α, in the airspace and plasma of patients with *P*. *aeruginosa*-induced acute respiratory distress syndrome. Thus, IL-36γ and its receptor signal not only impaired bacterial clearance in a possible PGE2 dependent fashion but also mediated lung injury during *P*. *aeruginosa* infection.

## Author summary

Pneumonia caused by *Pseudomonas aeruginosa* is a serious infection resulting in significant lung injury and mortality in susceptible hosts. The first line of defense in *P*. *aeruginosa* lung infection are neutrophils and macrophages, which play a pivotal role in the rapid clearance of pathogens from the lung. However, excessive innate responses against microbial pathogens can promote collateral damage to lung tissue, culminating in acute lung injury. Here, we demonstrated the role of IL-36 cytokines in modulating the innate immune response to *P*. *aeruginosa* pulmonary infection. The elaboration of interleukin (IL)-36γ in the alveolar space was observed not only in mice during *P*. *aeruginosa* pulmonary infection but also patients with pneumonia due to *P*. *aeruginosa*. In murine *P*. *aeruginosa* lung infection, deletion of IL-36γ or its receptor resulted in improved bacterial clearance associated with reduced prostaglandin E2 production, and attenuated lung injury independent of changes in leukocyte influx. Taken together, blockage of IL-36γ and its receptor signal may represent a viable immunomodulatory therapeutic approach in cytotoxic pseudomonas respiratory infection.

## Introduction

*Pseudomonas aeruginosa* is a Gram-negative bacterium that causes acute nosocomial infection as well as chronic infection in immunocompromised hosts. Infection with *P*. *aeruginosa* can lead to sepsis, pneumonia, and lung injury, which are often severe and life threatening [1]. Poor clinical outcomes in this disease are believed to be due to virulence factors expressed by this pathogen, increasing rate of multidrug resistance in *P*. *aeruginosa*, and the immune status of the infected hosts [2, 3].

Interleukin (IL)-36 cytokines, including three agonists IL-36α, IL-36β, IL-36γ and an antagonist IL-36Ra, are recently described members of IL-1 family of cytokines. IL-36 agonists bind the same receptor complex, consisting of the IL-36 receptor (IL-36R) and IL-1 receptor accessory protein (IL-1RAcP), which is shared with the IL-1 receptor and the IL-33 receptor [4]. IL-36R agonists are expressed by stimulated immune cells, such as monocytes and macrophages, dendritic cells, and epithelial cells [5–7]. The IL-36 receptor ligands induce pro-inflammatory cytokine and chemokine expression and contribute to neutrophil accumulation, dendritic cell activation and polarization of T helper 1 and IL-17 producing T cells [4, 8, 9]. Intratracheal administration of IL-36α or IL-36γ in mice induces a rapid influx of neutrophils into the lungs and pro-inflammatory cytokines and chemokines [10, 11]. Innate immune cell recruitment and phagocytic bacterial clearance, including neutrophils and macrophages, have critical roles in the host defense during the early stage of *P*. *aeruginosa* infection [12, 13]. Moreover, IL-36γ mRNA is upregulated in human bronchial cells after infection with *P*. *aeruginosa* [14]. Taken together, these observations suggest that IL-36 cytokines may play an important role in host defense against *P*. *aeruginosa*, perhaps by contributing to inflammatory cell recruitment/activation during infection, To date, the role of IL-36 cytokines in the host defense of *P*. *aeruginosa* infection has not been defined.

Prostaglandin E2 (PGE2) is a major product of arachidonic acid metabolism and its production is dependent on the cyclooxygenases (COX-1 and COX-2). Whereas COX-1 is expressed constitutively in most of cells and believed to be required for immune homeostasis, COX-2 is primarily an inducible enzyme that is expressed in response to stimulation by pro-inflammatory cytokines (e.g. TNF-α and IL-1β), microbial pathogens, and endogenously produced growth factors [15]. Cytotoxic strains of *P*. *aeruginosa* induce the production of PGE2 in pulmonary macrophages through COX-2 activation [16]. *In vitro* studies demonstrated that PGE2 can impair phagocytic properties and bactericidal activity of alveolar macrophages during *P*. *aeruginosa* infection [17, 18]. Conversely, COX-2 inhibition or genetic deletion have been shown to enhance bacterial clearance and reduce mortality in a murine *P*. *aeruginosa* murine model [16, 19]. No studies have assessed possible cross-talk between IL-36 cytokines and eicosanoids such as PGE2.

We hypothesized that IL-36 cytokines and their receptor; IL-36R regulate host mucosal immunity in acute *P*. *aeruginosa* lung infection. In this study, we demonstrate that IL-36γ produced by pulmonary macrophages (PMs) and alveolar epithelial cells (AECs) during *P*. *aeruginosa* lung infection promoted deleterious effects on host outcome. These deleterious effects appear to be mediated, in part, by IL-36γ-induced production of PGE2, resulting in impaired bacterial clearance, and IL-36γ-driven lung injury that occurred in a fashion independent of PGE2.

## Results

### IL-36 cytokines are selectively induced in the lungs during *P*. *aeruginosa* infection

**Fig 1:**
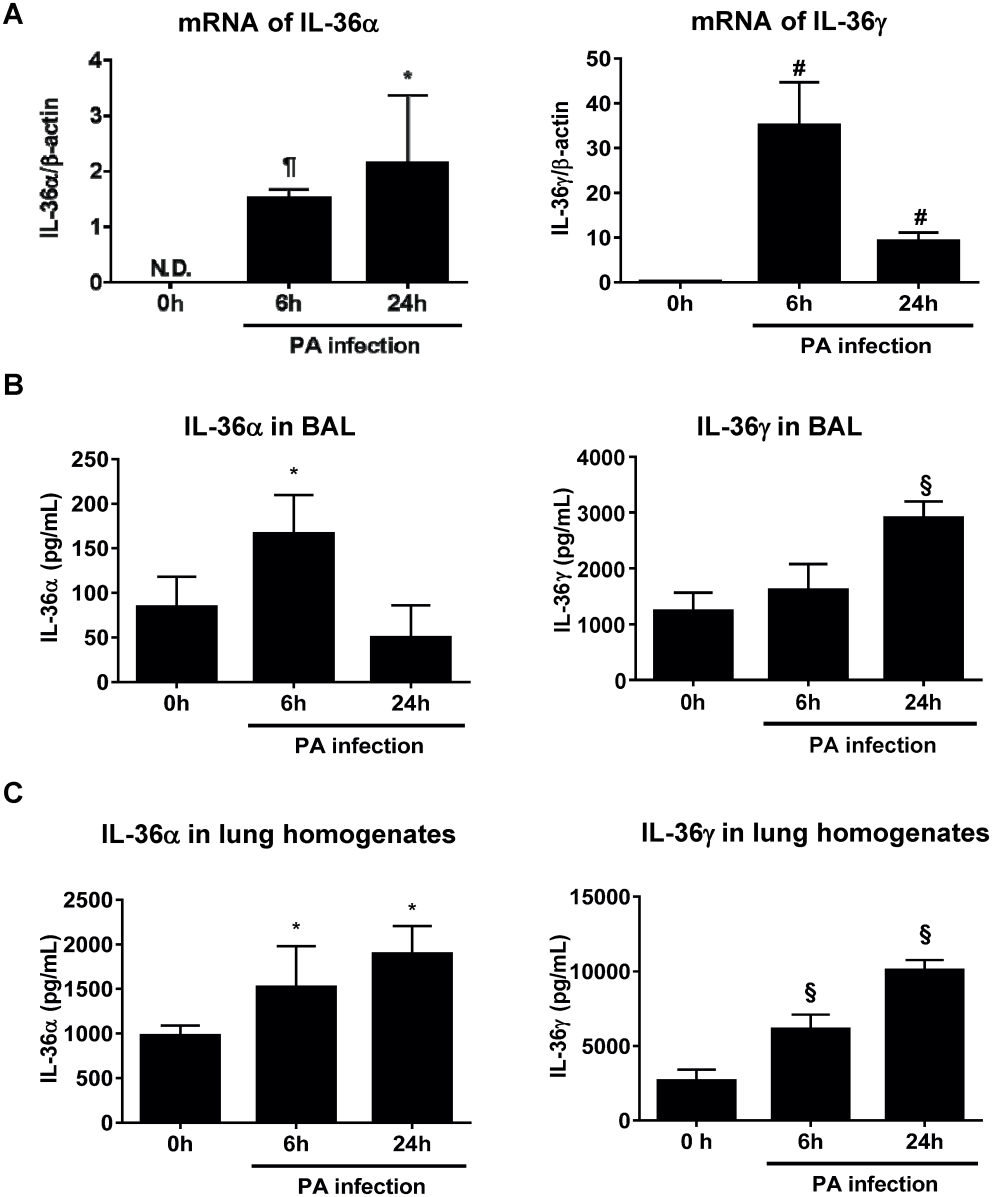
Expression of IL-36 cytokines in lung of WT mice infected with *P*. *aeruginosa*. Wild-type (WT) mice were infected intratracheally with 2.0 × 10^5^ colony forming unit (CFU) *P*. *aeruginosa*. (A) Transcript products of IL-36α (left panel) and IL-36γ (right panel) in the lungs of untreated, 6 h and 24 h after *P*. *aeruginosa* infection. mRNA was analyzed by real-time PCR. (B, C) Protein expression of IL-36α (left panel) and IL-36γ (right panel) in (B) bronchial alveolar lavage (BAL) and (C) lung homogenized tissue were quantified by ELISA. All data are shown as means ± SD of 4–5 mice/group. * *p*<0.05, # *p*<0.01, § *p*<0.001, significant compared with untreated mice. N.D.; not detected.

To examine whether IL-36 cytokines are expressed in *P*. *aeruginosa* lung infection, we first measured IL-36 cytokine mRNA in the lungs of wild-type (WT) C57B/6 mice infected with a flagellated cytoxic strain of *P*. *aeruginosa* (ATCC 19660). Both IL-36α and IL-36γ mRNA levels were significantly elevated in *P*. *aeruginosa* infected lungs at 6 h and 24 hrs post bacterial challenge (Fig 1A). No IL-36β mRNA was detected in *P*. *aeruginosa* infected lungs. Whereas the production of IL-36α in BAL fluid peaked at 4h and returned to baseline by 24 h after *P*. *aeruginosa* administration, IL-36γ levels in BAL fluid progressively increased at the 24 hr time point (Fig 1B). Similarly, both IL-36α and IL-36γ protein levels in homogenized lung tissue were increased in *P*. *aeruginosa* infection (Fig 1C). Considerably higher quantities of IL-36γ were found in BAL fluid and whole lung homogenates as compared to IL-36α (5–20 fold) Also, the expression of these cytokines was compartmentalized, as no IL-36α and IL-36γ was detected in plasma at these time points.

### IL-36α and IL-36γ are released from *P*. *aeruginosa* stimulated pulmonary macrophages and alveolar epithelial cells

**Fig 2:**
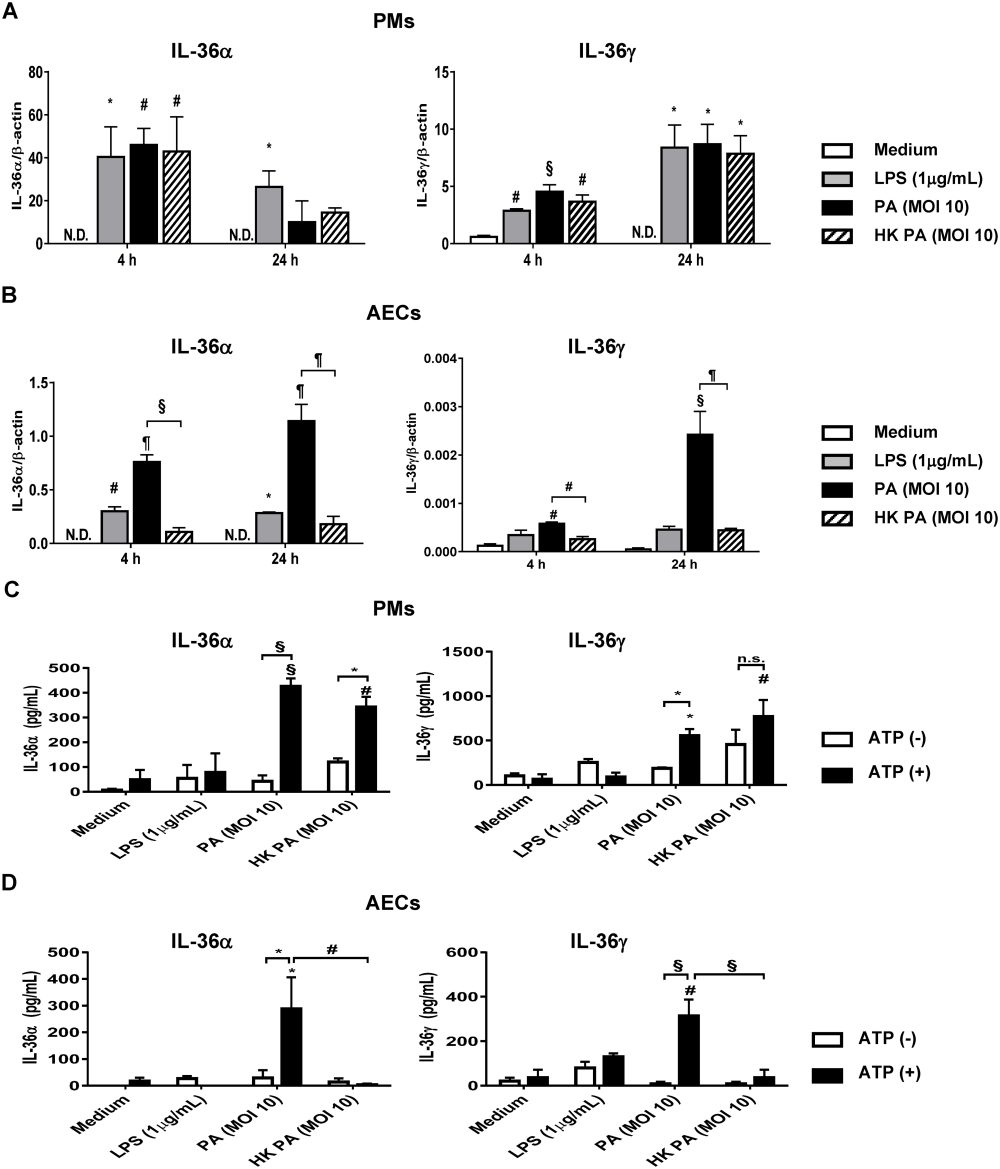
*P*. *aeruginosa* induced IL-36α and IL-36γ expression in primary pulmonary macrophages and alveolar epithelial cells. Primary pulmonary macrophages (PMs) and alveolar epithelial cells (AECs) isolated from WT mice were treated with LPS (1μg/ml), live *P*. *aeruginosa* or heat-killed *P*. *aeruginosa* at a multiplicity of infection (MOI) 10. (A, B) After 4 h and 24 h incubation, expression of IL-36α (left panel) and IL-36γ (right panel) mRNA in PMs (A) and AECs (B) were analyzed by real-time PCR. (C, D) After 24 h incubation, PMs and AECs were treated with or without ATP (5mM) during 20 min of incubation, and then culture medium (CM) were harvested. The protein levels of IL-36α (left panel) and IL-36γ (right panel) in CM of treated PMs (C) and AECs (D) were measured by ELISA. Data (means ± SEM) are representative of two independent experiments. N.D.; not detected. * *p*<0.05, # *p*<0.01, § *p*<0.001, ¶ *p*<0.0001, N.S.; not significant, compared with medium only or as indicated. LPS; lipopolysaccharide, PA; *Pseudomonas aeruginosa*, HK PA; heat killed *Pseudomonas aeruginosa*, ATP; adenosine triphosphate.

Respiratory epithelial cells and pulmonary macrophages are primary innate immune cells involved in pulmonary bacterial infection. To determine cellular source(s) of IL-36α and IL-36γ during *P*. *aeruginosa* infection, primary PMs and AECs isolated from WT mice were treated with lipopolysaccharide (LPS; 1μg/ml) or a multiplicity of infection (MOI) 10 of live or heat-killed *P*. *aeruginosa*. Significant induction of IL-36α and IL-36γ mRNA was observed in PMs in response to LPS, live or heat-killed *P*. *aeruginosa* as early as 4 h post stimulation, with persistent expression of IL-36γ mRNA level to 24h (Fig 2A). The expression levels of IL-36α and IL-36γ mRNA were significantly elevated in AECs treated with live *P*. *aeruginosa* at both 4 h and 24 h after stimulation (Fig 2B). While induction of IL-36α and IL-36γ mRNA levels was similar in PMs treated with either live or HK *P*. *aeruginosa*, live bacteria lead to much greater induction of IL-36α and IL-36γ mRNA in AECs, as compared to heat-killed bacteria (Fig 2A and 2B).

We next examined secretion of IL-36α and IL-36γ protein from *P*. *aeruginosa* treated-PMs and AECs. Previous studies indicated that extracellular adenosine triphosphate (ATP) [20, 21], or Caspase-3/7 activation [7, 22], were required for extracellular secretion of IL-36 cytokines, suggesting non-classical secretion mechanisms. Specifically, activation of the P2X7 receptor by ATP stimulation leads to changes in the morphology of lung epithelial cells [23] and macrophages [24], including plasma membrane blebbing, microvesicle release, and ultimately to apoptosis. Johnston et.al. demonstrated that secretion of IL-36γ in keratinocytes in response to bacterial flagellin was dependent on co-stimulation with ATP [21]. Marin et.al. observed that co-stimulation with LPS and ATP is necessary for IL-36α secretion from bone marrow-derived macrophages [20]. After 24 h in culture, ATP (5mM) was added, and conditioned medium was collected 20 min after ATP stimulation. In the absence of ATP, the secretion of IL-36α and IL-36γ into conditioned media (CM) was not elevated in LPS, live and heat-killed *P*. *aeruginosa*-treated PMs and AECs. Though we found a significant increase of IL-36α and IL-36γ production by PMs in response to live or heat-killed *P*. *aeruginosa* in combination with ATP (Fig 2C), only live bacteria plus ATP treatment increased the secretion of IL-36α and IL-36γ in AECs (Fig 2D). These data suggested PMs and AECs are likely cellular source of IL-36α and IL-36γ during *P*. *aeruginosa* lung infection.

Processing and secretion of IL-1β by macrophages in response to *P*. *aeruginosa* has been shown to require caspase-1 through activation of the inflammasome, triggered either by flagellin or type-III secretion system [25, 26]. Recently, we found that IL-36α mRNA expression in AECs is induced by influenza virus through caspase-1 activation dependent [7]. Moreover, other investigators have demonstrated that activation of caspase-1 by *Mycobacterium tuberculosis* enhanced IL-36γ mRNA in macrophages [27]. We next examined whether activation of caspase-1 contributed to the induction and secretion of IL-36α and IL-36γ by PMs in response to *P*. *aeruginosa*. We first confirmed that live *P*. *aeruginosa* treated-PMs upregulated the expression of capsae-1p10, and live, but not heat-killed, bacteria induced the production of IL-1β in CM by PMs (S1A and S1B Fig). Though caspase-1 inhibition attenuated the production of IL-1β by *P*. *aeruginosa*-treated PMs, *P*. *aeruginosa*-induced IL-36α and IL-36γ mRNA and protein expression were not altered by incubation with a caspase-1 inhibitor (S1C–S1E Fig). These data suggested that unlike influenza and tuberculosis infection, activity of caspase-1 was not involved in the induction and secretion of IL-36α and IL-36γ by PMs in response to *P*. *aeruginosa*. Of note, live *P*. *aeruginosa* did not increase activity of caspase-3/7 in either primary PMs or AECs (S2A and S2B Fig).

### IL-36R^-/-^ and IL-36γ^-/-^, but not IL-36α^-/-^ mice are protected during *P*. *aeruginosa* pneumonia

**Fig 3:**
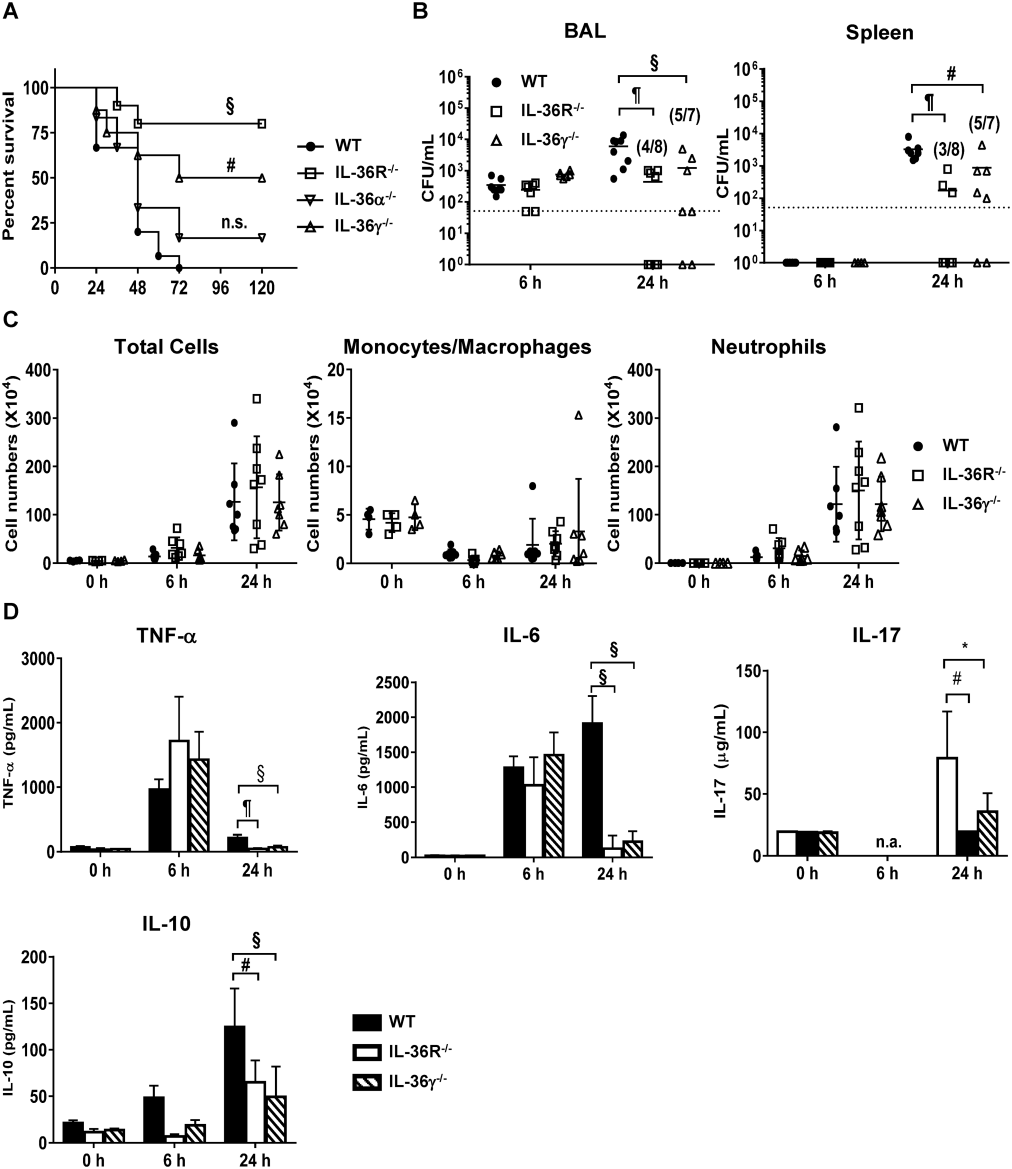
IL-36 receptor and IL-36γ deficient mice were resistant to acute *P*. *aeruginosa* infection. WT, IL-36 receptor deficient (IL-36R^-/-^) and IL-36γ deficient (IL-36γ^-/-^) mice were intratracheally infected with 2.0 × 10^5^ CFU *P*. *aeruginosa*. (A) Survival rate were assessed every 12 h following *P*. *aeruginosa* infection. Each group consisted of 6–10 mice. Survival curves were analyzed using the log-rank (Mantel–Cox) test. (B) Bacterial burden in BAL (left upper) and bacterial dissemination in spleen (left panel) were assessed by subsequent dilution method (n = 6–8 per each group). 50 CFU/mL is the limit of detection. Horizon bar indicate mean values. (C) The number of total cell (left panel), neutrophils (middle panel) and monocytes and macrophages (right panel) in BAL were counted at 0 h, 6 h and 24 h after infection. Each group consisted of 4–8 mice. (D) The production of TNF-α, IL-6, IL-10 and IL-17 were measured by ELISA (n = 4–8 each group). (C, D) Data are shown as mean (B) or means ± SD. # *p*<0.01, § *p*<0.001, ¶ *p*<0.0001, n.s.:not significant, n.a.: not available, compared with infected in WT mice or as indicated.

To elucidate the functional role of IL-36 receptor ligands during *P*. *aeruginosa* pulmonary infection, WT mice, IL-36α deficient (IL-36α^-/-^), IL-36γ deficient (IL-36γ^-/-^), and IL-36 receptor (IL-36R^-/-^) mice were challenged intratracheally with 2 × 10^5^ colony forming unit (CFU) *P*. *aeruginosa* and survival assessed (Fig 3A). Whereas all WT mice were dead within 72 h after bacteria challenge, 80% of IL-36R^-/-^ mice were long term survivors. Importantly, the survival rate of infected IL-36γ^-/-^ mice (50% survival) was also significantly increased compared to WT mice, and was not significantly different with that of IL-36R KO mice. By comparison, the survival rate of infected IL-36α^-/-^ mice was less than 20% and was not statistically different than WT mice. We omitted IL-36α^-/-^ mice from subsequent experiments based on lesser expression of IL-36α relative to L-36γ and lack of survival benefit in infected IL-36α^-/-^ mice.

We next examined whether the increased survival rate of *P*. *aeruginosa* infected IL-36R^-/-^ and IL-36γ^-/-^ mice was associated with differences in bacterial clearance and dissemination post *P*. *aeruginosa* challenge. Bronchoalveolar lavage (BAL) and homogenized spleen samples were obtained to determine bacterial loads at 6 h and 24 h after *P*. *aeruginosa* infection. At 6 h post bacterial challenge, *P*. *aeruginosa* CFU in BAL were similar among WT mice, IL-36R^-/-^ mice and IL-36γ^-/-^ mice. By 24 h, bacteria CFU in BAL were approximately10- and 5-fold lower in IL-36R^-/-^ and IL-36γ^-/-^ mice than in WT mice, respectively (Fig 3B, left panel). Moreover, reduced bacterial dissemination, as assessed by splenic CFU, was observed in IL-36R^-/-^ and IL-36γ^-/-^ mice at 24 h after bacterial administration, as compared to their WT counterparts (Fig 3B, right panel).

We next quantified differences in lung inflammatory cell accumulation in WT mice, IL-36R^-/-^ mice and IL-36γ^-/-^ mice during *P*. *aeruginosa* infection. No difference in number of total BAL leukocyte and proportion of monocytes/macrophages were observed in uninfected WT mice, IL-36R^-/-^ mice and IL-36γ^-/-^ mice at baseline. Greater than 90% of leukocytes in *P*. *aeruginosa*-infected WT mice were neutrophils at 6 h and 24h. Interestingly, we did not find any differences in the number of total cells, neutrophils and monocytes/macrophages among three groups at the selected time points examined during *P*. *aeruginosa* infection (Fig 3C).

Cytokines and chemokines are important role in host immunity and as mediators of collateral lung injury during experimental *P*. *aeruginosa* pulmonary infection [28]. To examine whether IL-36 receptor and IL-36γ genetic deletion altered the production of pro-inflammatory and anti-inflammatory cytokines during *P*. *aeruginosa* infection, we measured the levels of TNF-α, IL-6, and IL-10 in BAL fluid. At 6 h post *P*. *aeruginosa*, the levels of these mediators were similar among the three groups. However, at 24 h after *P*. *aeruginosa* administration, TNF-α, IL-6 and IL-10 were significantly lower in BAL fluid from IL-36R^-/-^ and IL-36γ^-/-^ mice as compared with WT mice (Fig 3D). We also found significantly lower levels of IL-17 in the BAL fluid of mutant mice at 24 hrs post infection (Fig 3D). IL-17 has been shown to play a critical role in the innate response against extracellular bacterial pathogens, in part through regulating PMN influx and antimicrobial peptide expression. However, there was no difference in BAL PMN accumulation (Fig 3C) and mRNA level of β-defensin3 and cathelicidin antimicrobial peptide (CAMP) among three groups (S3 Fig).

### IL-36R^-/-^ and IL-36γ^-/-^ mice are protected from lung injury during *P*. *aeruginosa* infection

**Fig 4:**
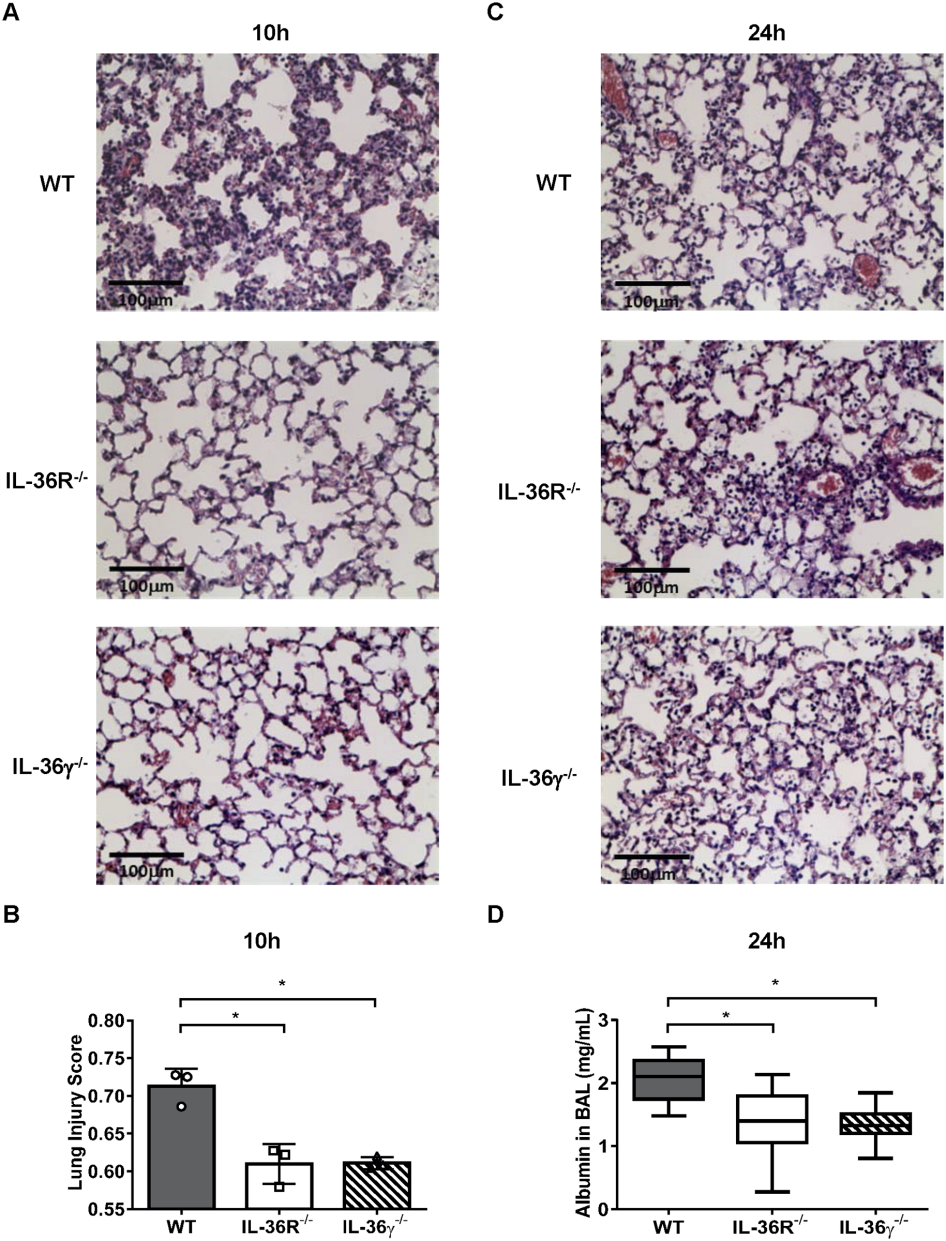
IL-36 receptor and IL-36γ deficient mice attenuated lung injury during *P*. *aeruginosa* infection. (A, C) Lung histopathological analysis in *P*. *aeruginosa* infected WT, IL-36R^-/-^ and IL-36γ-/- mice was performed at 10 h (A) and 24 h (C) post infection. H&E-stained lung tissue at magnification of 400X. (B) The quantification of lung injury in lung sections with infected-WT, IL-36R^-/-^ and IL-36γ^-/-^ mice at 10 h were evaluated as previously described (n = 3 per each group). Lung injury scoring system parameters include neutrophils in the alveolar space (i), neutrophils in the interstitial space (ii), hyaline membranes (iii), proteinaceous debris filling the airspaces (iv) and alveolar septal thickening (v). At least 20 random regions were scored 0–2 independently and the final lung injury score was calculated as below; score = [(20 × i) + (14 × ii) + (7 × iii) + (7 × iv) + (2 × v)] / (number of fields × 100). (D) Lung permeability was quantified by albumin concentration in BAL fluid from 6 h and 24 h post infection (n = 6–8 per each group). All data are shown as means ± SD. * *p*<0.05, compared with WT mice.

To examine the role of IL-36 ligands in *P*. *aeruginosa* induced lung injury, we performed semi-quantitative analysis of lung histology slides from WT mice, IL-36R^-/-^ mice and IL-36γ^-/-^ mice at 10 h and 24 h after *P*. *aeruginosa* infection. At 10 h, *P*. *aeruginosa*-infected lung of WT mice showed significant histological abnormalities, including alveolar wall edema and inflammatory cells accumulation in the lung interstitium, reflective as a lung injury score of 0.71. In contrast, at this point, *P*. *aeruginosa* infected lung histology in IL-36R^-/-^ mice and IL-36γ^-/-^ mice displayed significantly less edema and epithelial cell disruption than that in WT mice (Fig 4A and 4B). At 24, there was substantial infiltration of neutrophils within the intraalveolar septa and alveolus, as well as alveolar septa edema and proteinaceous debris in the alveolar space, with a similar histological pattern observed in all three groups (Fig 4C). We next assessed the integrity of the alveolar-capillary membrane by measurement of albumin concentration in BAL fluid. At 24 h post infection, albumin levels in BAL fluid were significantly reduced in IL-36R^-/-^ mice and IL-36γ^-/-^ mice compared to WT mice (Fig 4D).

### IL-36γ induced COX-2 expression and the production of PGE2 in PM *in vitro*

**Fig 5:**
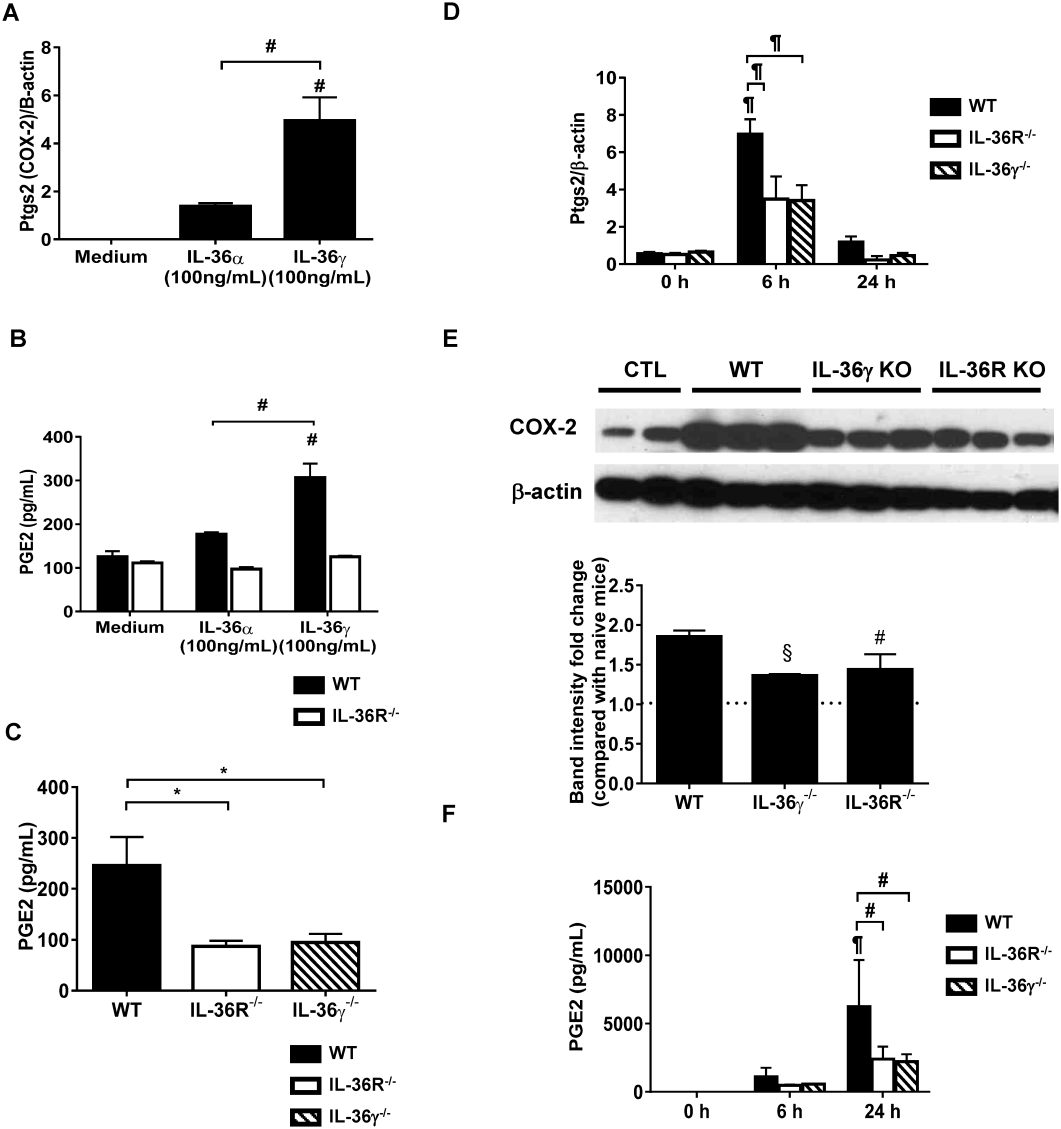
IL-36γ induced COX-2 mRNA expression and the production of PGE2 in PMs during *P*. *aeruginosa* infection. (A-C) Primary PMs isolated from WT and IL-36R^-/-^ mice were treated with recombinant IL-36α (100ng/ml) and IL-36γ (100ng/ml) for 24 h. (A) The expression of prostaglandin-endoperoxide synthase 2/cyclooxygenase 2 (Ptgs2/COX-2) mRNA in PMs was analyzed by real-time PCR. (B) The protein production of prostaglandin e 2 (PGE2) in CM by PMs was examined by ELISA. (C) PMs isolated from WT, IL-36R^-/-^ and IL-36γ^-/-^ mice were treated with live *P*. *aeruginosa* at a MOI 10 for 24 h. (C) PGE2 production in CM were examined. Data (means ± SEM) are representative of two independent experiments. * *p*<0.05, # *p*<0.01, significant compared with medium only or as indicated. (D-F) WT, IL-36R^-/-^ and IL-36γ^-/-^ mice were intratracheally infected with 2.0 × 10^5^ CFU *P*. *aeruginosa*. (D) PTGS2 mRNA and (F) the production of PGE2 in BAL in infected-WT, IL-36R^-/-^ and IL-36γ^-/-^ mice were examined (n = 4 per each group). (F) The expression of COX-2 in the lungs was determined by Western blotting. Band intensity represents relative density compared with β-actin, and fold changes compared with the lungs of naïve WT mice were presented. # *p*<0.01, ¶ *p*<0.0001, significant compared with untreated mice or as indicated.

PGE2 is known to be an important lipid mediator with a variety of immunosuppressive properties. Previous studies suggest that PGE2 mediates impaired bacterial clearance during *P*. *aeruginosa* infection [16, 18, 19]. Thus, we first examined the effect of IL-36 receptor ligands on regulating PGE2 production in primary PMs. PMs isolated from WT mice were stimulated with recombinant IL-36α (100 ng/mL) and IL-36γ (100 ng/mL) for 24 h. The expression level of prostaglandin-endoperoxidase synthase 2 (Ptgs2) mRNA was significantly elevated in rIL-36-treated PMs, with rIL-36γ being a more potent inducer of Ptgs2 than rIL-36α (Fig 5A). Induction of Ptgs2 occurred in a dose dependent fashion, as no expression of Ptgs2 mRNA was observed in PMs which was treated with 1 ng/ml or 10 ng/ml concentrations of rIL-36γ. Treatment with rIL-36γ, but not rIL-36α, significantly induced the production of PGE2 in PMs isolated from WT mice. This induction was dependent upon IL-36R signaling, as no induction of PGE2 was observed in PMs isolated from IL-36R^-/-^ mice (Fig 5B), excluding an off target effect of rIL-36γ.

We next examined whether autocrine secretion of IL-36 receptor ligands induced by *P*. *aeruginosa* stimulation regulated COX-2 expression and PGE2 production in PMs. PMs isolated from WT mice, IL-36R^-/-^ mice and IL-36γ^-/-^ mice were stimulated with *P*. *aeruginosa* at a MOI of 10 for 24 h. The production of PGE2 was significantly higher in *P*. *aeruginosa*-treated PMs from WT mice than in that from IL-36R^-/-^ mice and IL-36γ^-/-^ mice (Fig 5C). These data suggest that PGE2 synthesis by lung macrophage may be dependent upon autocrine and paracrine IL-36γ secretion in response to *P*. *aeruginosa*.

We also examined the possibility that AECs could be an important cellular source of PGE2 production in response to rIL-36α (100ng/ml) or rIL-36γ (100ng/ml). Although the treatment with rIL-36γ induced Ptgs2 mRNA in AECs isolated from WT mice, we did not observe PGE2 production by AECs treated with either rIL-36γ or rIL-36α (S4A and S4B Fig).

### IL-36γ–induced PGE2 impaired bacterial clearance during *P*. *aeruginosa* infection *in vivo*

Next, we examined whether autocrine or paracrine IL-36γ and its receptor; IL-36R contributed to PGE2 synthesis during *P*. *aeruginosa* infection *in vivo*. *P*. *aeruginosa* markedly increased the expression of Ptgs2 mRNA in the lung of WT mice at 6 h after infection, whereas we observed significantly lower expression of Ptgs2 mRNA in infected IL-36R^-/-^ mice and IL-36γ^-/-^ mice (Fig 5D). We next examined whether IL-36γ and its receptor contributed to COX-2 protein expression in the lungs during *P*. *aeruginosa* infection. Infected lungs of IL-36γ^-/-^ and IL-36R^-/-^ mice showed significantly lower expression of COX-2 compared with infected WT mice by Western blot analysis (Fig 5E). In addition, the production of PGE2 in BAL at 24 h was significantly attenuated in IL-36R^-/-^ mice and IL-36γ^-/-^ mice as compared to infected WT animals (Fig 5F).

**Fig 6:**
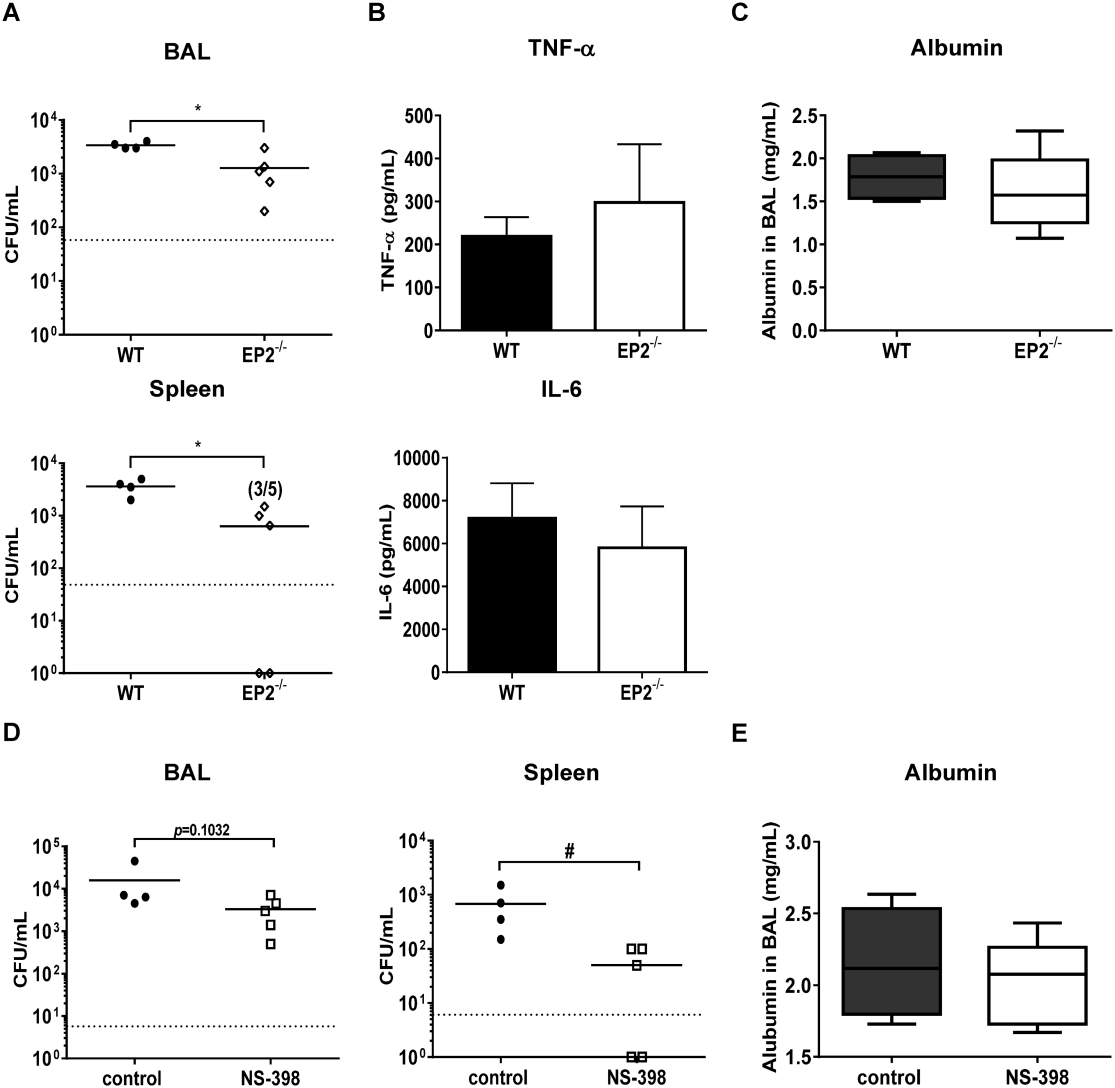
Impact of PGE2-EP2 signal in *P*. *aeruginosa* infection *in vivo*. (A-C) WT and PGE2 receptor 2 deficient (EP2^-/-^) mice were intratracheally infected with 2.0×10^5^ CFU *P*. *aeruginosa*. After 24 h infection, (A) bacterial counts in BAL (upper panel) and homogenized spleen samples (lower panel), (B) the production of TNF-α and IL-6 in BAL and (C) the albumin concentration in BAL were examined. Each group consisted of 4–5 mice. (D, E) WT mice were administrated COX-2 inhibitor (NS398; 10μM) or vehicle intraperitoneally 1 h prior to 2.0 × 10^5^ CFU *P*. *aeruginosa* challenge. (D) Bacterial counts in BAL (left panel) and homogenized spleen samples (right panel), (E) the albumin concentration in BAL were examined. Each group consisted of 4–5 mice. Data are shown as mean (A, D) or means ± SD (B, C and E). * *p*<0.05, # *p*<0.01, ¶ *p*<0.0001, significant compared with untreated mice or as indicated.

To confirm the impact of PGE2 on host defense against *P*. *aeruginosa* pulmonary infection, we used PGE2-receptor subtype 2 (EP2) receptor deletion mice, as EP2 is a major receptor responsible for the immunosuppressive and anti-inflammatory properties of PGE2 [29]. Previous study demonstrated that the profile of EP receptor in PMs is EP2>EP1>EP4>EP3 and activated macrophages increased the expression of EP2 and decreased EP4 expression [18]. WT mice and EP2^-/-^ mice were treated intratracheally with 2.0 × 10^5^ CFU *P*. *aeruginosa*, and then quantitated bacterial burden in BAL and spleen, the production of pro-inflammatory cytokines and albumin concentration in BAL at 24 h post infection (Fig 6A–6C). Bacterial loads in BAL and spleen in EP2^-/-^ mice were 2.7- and 5.8-fold lower compared to WT mice, respectively (Fig 6A). However, we did not find differences in levels of TNF-α and IL-6 (Fig 6B) or albumin concentration (Fig 6C) in BAL between infected WT mice and EP2^-/-^ mice. Finally, to confirm contribution from the COX-2/PGE2 pathway in host defense against *P*. *aeruginosa* pneumonia, we administrated the COX-2-specific inhibitor NS-398 (10μM) to WT mice i.p. 1 h before bacterial challenge. As shown Fig 6D and 6E, treatment with NS-398 result in a trend toward to reduce the bacterial CFU in BAL and significantly reduced the dissemination in *P*. *aeruginosa* infected mice. Similar to observations in EP2^-/-^ mice, we observed no difference in BAL albumin concentration between treatment with NS-398 and vehicle control. These data suggest that COX2/PGE2/EP2 signaling in this model can promote impaired antimicrobial immunity but does not appear to regulate inflammatory cytokine production or lung injury responses.

### IL-36γ-induced PGE2 attenuated PM bacterial killing *in vitro*

**Fig 7:**
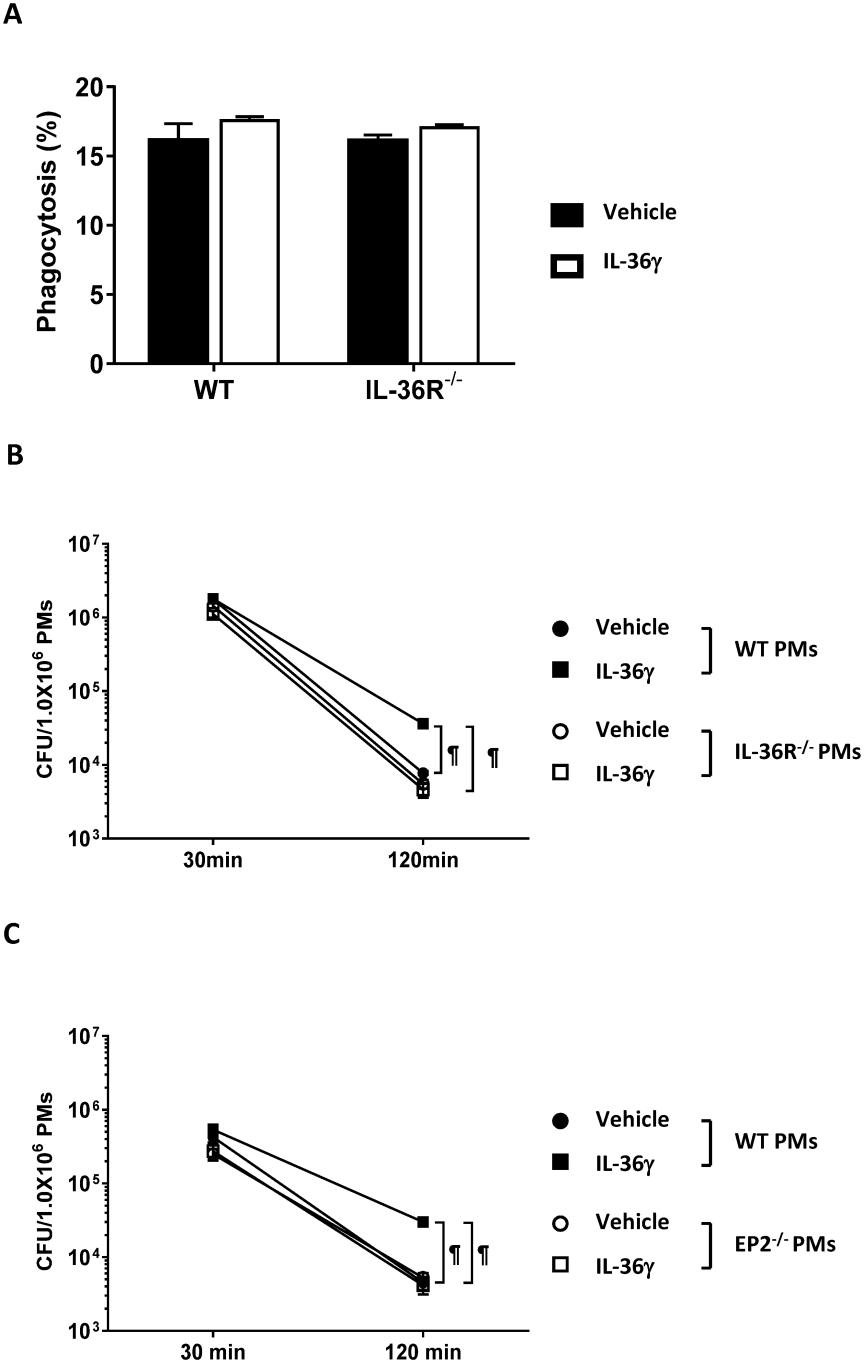
IL-36γ impairs PM bacterial killing during *P*. *aeruginosa* and dependence on PGE2-EP2 signal. Primary PMs were harvested from WT, IL-36R^-/-^ or EP2^-/-^ mice, and treated with or without recombinant IL-36γ (100ng/ml) for 18h. (A) Vehicle or rIL-36γ treated PMs (1 × 10^6^ cells/well) isolated from WT and IL-36R-/- mice were incubated with for 2 h with FITC-labelling heat killed *P*. *aeruginosa* at a MOI 100. After 2h incubation, cells were collected and analyzed the phagocytic response as FITC positive cells by flow cytometry. Data showed the percentage of phagocytic PMs. (B) Vehicle or rIL-36γ treated PMs (1 × 10^6^ cells/well) from WT and IL-36R^-/-^ mice, (C) Vehicle or rIL-36γ treated PMs (1 × 10^6^ cells/well) isolated from WT and EP2^-/-^ mice were incubated with live *P*. *aeruginosa* at a MOI 100. PMs were harvested at 30 min to quantify CFU as the initial time point, or incubated further for an additional 90 min. CFU/10^6^ PMs were obtained each samples by subsequent dilution method. Data (means ± SEM) are representative of two independent experiments. * *p*<0.05, § *p*<0.001, ¶ *p*<0.0001, compared with untreated mice or as indicated.

We next examined whether IL-36γ directly regulated *P*. *aeruginosa* phagocytosis and bacterial killing by PMs, PMs were isolated from WT mice and IL-36R^-/-^ mice, then treated with rIL-36γ (100ng/ml) for 18h, then cells incubated with FITC-labeled heat-killed *P*. *aeruginosa*. PMs isolated from WT and IL-36R^-/-^ mice showed similar phagocytosis of FITC-labeled bacteria. Additionally, treatment with rIL-36γ did not alter the phagocytic ability of PMs isolated from WT mice (Fig 7A). For bacterial killing assays, PMs were incubated with live PA, washed, and viable intracellular CFU determined by standard culture techniques. The number of viable intracellular bacteria in PMs isolated from WT mice and IL-36R^-/-^ mice was similar at 30 min and 120 min after *P*. *aeruginosa* inoculation. Interestingly, IL-36γ treated PMs from WT mice displayed impaired bactericidal activity, as evident by a significantly higher number of viable bacteria at 120 min post bacterial inoculation than non-treated PMs (Fig 7B). This effect was dependent on specific IL-36 receptor signaling, as rIL-36γ treatment did not alter bacterial killing in PMs isolated from IL-36R^-/-^ mice.

To examine whether PGE2 might be responsible for IL-36γ- mediated impairment in microbicidal activity, we isolated PMs from WT mice and mice lacking the EP2 receptor. Killing of intracellular bacteria by PMs isolated from EP2^-/-^ mice was similar to PMs isolated from WT mice. However, Treatment of WT PM with rIL-36γ resulted in decreased microbicidal activity, whereas incubation with rIL-36γ did not inhibit microbicidal activity in PMs lacking EP2 (Fig 7C). In addition, we examined whether IL-36γ-induced COX-2 mediated impaired bacterial killing by PMs. Treatment with NS-398 enhanced bacterial killing activity in IL-36γ treated PMs (S5E Fig). Thus, IL-36γ-induced impairment in PM microbicidal activity requires COX-2/PGE2/EP2 signaling.

### IL-36γ is increased in the airspace and plasma of patients with *P*. *aeruginosa*-induced acute respiratory distress syndrome

**Fig 8:**
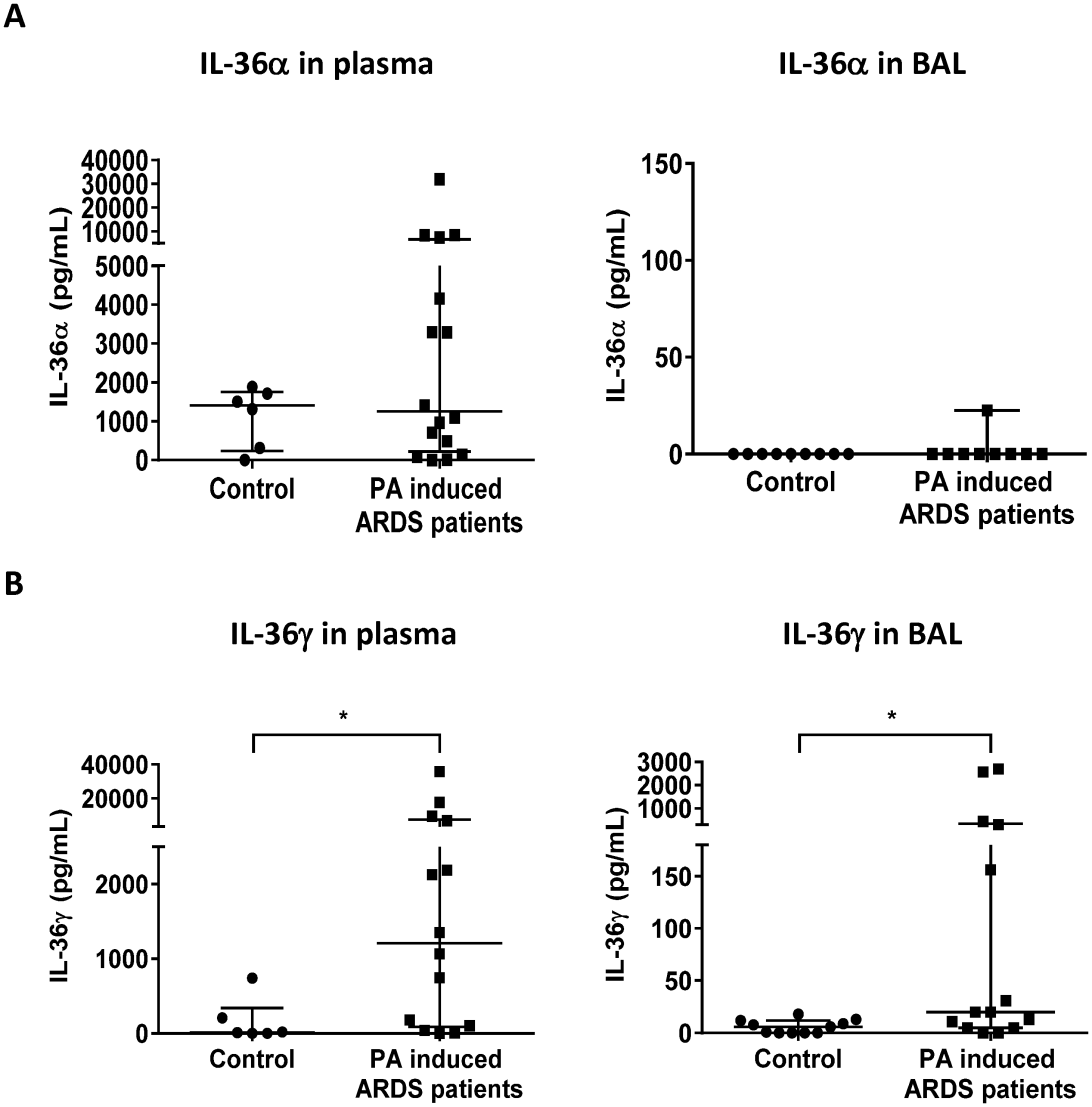
Elevated IL-36γ in patients with *P*. *aeruginosa* induced ARDS. Plasma and BAL samples were obtained from acute respiratory distress syndrome (ARDS) patients caused by *P*. *aeruginosa* from day 0 to day 7 after onset and healthy control. The levels of (A) IL-36α and (B) IL-36γ in plasma and BAL were measured by ELISA. Sample number: healthy control (plasma; n = 6 and BAL; n = 9), patients with *P*. *aeruginosa* induced ARDS (plasma; n = 16 and BAL; n = 10). All data were showed as median with interquartile range. * *p*<0.05 compared with healthy control.

Finally, to determine whether observations in *P*. *aeruginosa* murine pneumonia model were of potential clinical relevance, we measured levels of IL-36α and IL-36γ in plasma and BAL fluid of patients with acute respiratory distress syndrome (ARDS) caused by *P*. *aeruginosa* within 7 days from the onset of ARDS. We found that IL-36γ were significantly elevated in both plasma and BAL samples of patients with *P*. *aeruginosa* induced ARDS patients, as compared to healthy subjects (Fig 8A and 8B). By comparison, a trend toward elevated IL-36α levels was found in plasma but not BAL of these patients, as compared to healthy subjects.

## Discussion

In Gram-negative bacterial pneumonia, the innate immune system, including neutrophils and macrophages, plays a necessary role in the rapid clearance of pathogens from the lung. However, a vigorous inflammatory response against microbes, particular pathogens that express a broad armament of virulence factors, can promote collateral damage to lung tissue resulting in acute lung injury [30]. In this study, we demonstrate that IL-36γ released from PMs, AECs and likely other cellular source is not only dispensable for bacterial clearance, but can actually inhibit antimicrobial immunity, an effect that is at least partially PGE2-dependent. Moreover, IL-36 ligand can promote lung injury by a mechanism that is likely independent of PGE2.

Stimulation with bacteria and virus induces IL-36 cytokines from epithelial cells, macrophages and monocytes in the lungs [6, 7, 27]. We have recently shown that influenza virus stimulates production of IL-36α by AECs, and IL-36 receptor deficient mice prevent from influenza virus-induced lung injury [7]. However, current knowledge of the functional role of IL-36 receptor ligands in bacterial infection is limited. We found significantly increased level of IL-36α and IL-36γ in the lungs of *P*. *aeruginosa* infected mice (Fig 1), and *P*. *aeruginosa*-treated PMs and AECs expressed IL-36α and IL-36γ (Fig 2). Importantly, the functional phenotype IL-36γ^-/-^ mice during infection resembled that of IL-36R^-/-^ mice, whereas survival of IL-36α^-/-^ mice paralleled that of infected WT mice (Fig 3A). We cannot exclude a minor contribution of IL-36α to the phenotype observed, but IL-36γ appears to be predominant IL-36R agonist responsible for the observed effects. The quantity of IL-36α in *P*. *aeruginosa* infected lungs was considerably less than that of IL-36γ production. *In vitro*, treatment with rIL-36γ, but not IL-36α significantly induced COX-2 mRNA expression and the production of PGE2 in PMs. Findings in the murine model are in keeping with our observations in patients with Pseudomonas pneumonia and ARDS, as we observed significantly higher levels of IL-36γ, but not IL-36α, in plasma and BAL as compared with healthy control subjects (Fig 8). Collectively, these data suggest that IL-36γ is the predominant IL-36R ligand involved in the pathogenesis of *P*. *aeruginosa* induced severe pneumonia. Of note, all patients with Pseudomonas pneumonia also had ARDS, so we were unable to distinguish the influence of pseudomonal infection alone from that of the immune landscape of acute lung injury.

A somewhat unanticipated finding was the observation that IL-36R^-/-^ mice and IL-36γ^-/-^ mice displayed decreased bacterial load in the alveolar space and less dissemination during *P*. *aeruginosa* infection (Fig 3B). This is unlikely to be a direct effect of IL-36 agonists on neutrophil accumulation and activation, as we observed no differences in PMN numbers and mouse neutrophils do not express IL-36 receptor [31, 32]. Furthermore, we did not observe differences in phagocytosis and bacterial killing in neutrophils isolated from WT mice, IL-36R^-/-^ mice and IL-36γ^-/-^ mice during *P*. *aeruginosa* infection *in vitro* (S5A and S5B Fig). Also, there were no differences in phagocytic and microbicidal activity of PM isolated from WT mice, IL-36R^-/-^ mice and IL-36γ^-/-^ mice (S5C and S5D Fig and Fig 7A and 7B). These data implicate other host derived mediators induced by IL-36γ that contribute to impaired bacterial clearance. It is worth noting that there was a more impressive reduction in bacterial dissemination (as reflected by splenic CFU) in IL-36R^-/-^ mice and IL-36γ^-/-^ mice when compared to WT animals than reductions in BAL CFU observed in these animals, which is more modest. We believe this is attributable to less lung injury and disruption of the alveolar-capillary membrane in the mutant mice post *P*. *aeruginosa* challenge.

Our findings implicate PGE2 as a relevant contributor to IL-36-mediated suppression of anti-pseudomonal immunity. PGE2 is a well-recognized lipid regulator of inflammatory and immune responses during acute and chronic infections. IL-1β, a family member of IL-36 cytokines, is known to induce COX-2 expression and production of PGE2 in macrophages [33, 34], and the induction of macrophage-derived PGE2 in response to *Mycobacterium tuberculosis* is dependent upon IL-1 receptor ligands [35]. However, interactions between IL-36 receptor ligands and PGE2 expression have not previously been described. We found that rIL-36γ, but not rIL-36α, dose-dependently induced Ptgs2/COX-2 mRNA expression and PGE2 production in PMs, and that COX-2 expression and PGE2 production by PMs isolated from IL-36R^-/-^ mice and IL-36γ^-/-^ mice *in vitro* were impaired relative to PMs isolated from WT mice (Fig 5). Vigne et.al. demonstrated that rIL-36β the induction of IL-6, CXCL-1, CCL1 and IL-23p19 mRNA by rIL-36β was 1.5–2.0 fold greater than that induced by rIL-36α and IL-36β in bone marrow derived dendritic cells [31] *in vitro*. Other investigator have shown that rIL-36γ, but not rIL-36α significantly induced TNF-α, and only IL-36β could induce β-defensin (HBD)-2, HBD-3 and CAMP in human keratinocytes [21]. Collectively, these studies suggest that response to IL-36 cytokines is depends on both the specific IL-36 ligand and the cells which expresses IL-36 receptor. In addition, the i*n vivo* expression of COX-2 and production of PGE2 in the lungs of *P*. *aeruginosa*-infected IL-36R^-/-^ mice and IL-36γ^-/-^ mice was mitigated as compared to infected WT animals (Fig 6B and 6C). Importantly, the defect in PGE2 production was similar in IL-36R^-/-^ mice and IL-36γ^-/-^ mice. Taken together, endogenously produced IL-36γ and its receptor signal contribute to the induction of PGE2 during *P*. *aeruginosa* infection.

Previous studies have demonstrated a relevant immunomodulatory influence of prostaglandins on antimicrobial function of phagocytes, including PMN and mononuclear phagocytes. For instance, activation and aggregation of neutrophils is inhibited after exogenous treatment with PGE2 *in vitro* [36, 37]. In addition, PGE2 impaired the ability of PMN to kill *P*. *aeruginosa* [18]. In other murine *P*. *aeruginosa* infection models, PGE2 has been shown to impair both internalization and killing of ingested bacteria by macrophages [18, 17]. In our study, we did not find differences in phagocytic properties (Fig 7A) and initial intracellular bacterial loads in PMs (Fig 7B) between IL-36γ and vehicle treated PMs *in vitro*. In addition, no significant difference in BAL bacterial CFU was observed among infected WT mice, IL-36R^-/-^ mice and IL-36γ^-/-^ mice at early period post infection (6 hrs, Fig 3B) *in vivo*. These data indicates that IL-36γ does not contribute to initial bacteria uptake by resident leukocytes. Reactive oxygen species and nitric oxide (NO) are important mediators of macrophages bactericidal activity in *P*. *aeruginosa* infection [38, 39]. PGE2 has been shown to suppress NO synthesis in murine macrophages [15], and microbicidal activity in phagocytic cells [38, 39]. We found impaired bacterial killing in PMs treated with IL-36γ, and this effect was mitigated in PMs isolated from EP2 receptor deficient mice (Fig 7B and 7C). In addition, EP2^-/-^ mice showed decreased bacterial loads in BAL and attenuated dissemination in spleen compared with WT mice during *P*. *aeruginosa* infection (Fig 6C), findings that mirrored that observed in both IL-36γ^-/-^ and IL-36R deficient mice. While these observations point to PGE2 as a major mediator of impaired antimicrobial immunity in IL-36γ/IL-36R mutant mice, there are likely other mediators involved. One such candidate is IL-10, which can suppressive antimicrobial responses and the in-vivo production of IL-10 was reduced in both infected IL-36R^-/-^ mice and IL-36γ^-/-^ mice. However, inhibition of IL-10 bioactivity by neutralizing antibody administration did not alter bacterial clearance in our model (S6 Fig), suggest that IL-10 was not responsible for the effects observed.

The finding of improved *P*. *aeruginosa* clearance and reduced dissemination in IL-36γ^-/-^ mice differs from observations we have recently made in another murine Gram-negative bacterial pneumonia model (Klebsiella pneumonia) [40]. In this model, we observed that IL-36γ^-/-^ mice were more susceptible to *Klebsiella pneumoniae* challenge due to impaired expression of type 1 and IL-17 cytokines. There may be several reasons for these disparate findings. First, *K*. *pneumonia*e is a heavily encapsulated organism that is much more invasive than *P*. *aeruginosa*, and a vigorous type 1 and IL-17 innate response is required for clearance from the airspace. By comparison, challenge with *P*. *aeruginosa*, especially cytotoxic strains such as ATCC 19660, results in marked and deleterious inflammation and injury, and invasion only occurs with high inoculum of organisms. Indeed, in pneumonia caused by Klebsiella pneumonia murine model, pro-inflammatory cytokines such as TNF-α, IL-1, and IL-17 [41, 42] are required for bacteria clearance from the lungs, whereas the anti-inflammatory cytokine IL-10 impairs host defense in this infection model [43]. Conversely, the effect of these pro-inflammatory and anti-inflammatory cytokine in the host defense against *P*. *aeruginosa* is opposite to that observed in *K*. *pneumoniae* infection [44–46]. Moreover, the peak of lung IL-36γ expression after bacteria challenge is earlier post *P*. *aeruginosa* administration as compared to *K*. *pneumoniae* administration, which may be partially attributable to the much higher inoculum used in the *Pseudomonas* model [40], We speculate that early and marked cytokine storm induced by cytotoxic strains of *P*. *aeruginosa* promotes deleterious lung injury, Moreover, PGE2 is highly induced and plays an important immunoregulatory role in *Pseudomonas* lung infection, whereas this has not been as convincingly shown in more progressive and invasive infections such as *K*. *pneumoniae*.

Lung injury was mitigated in IL-36R^-/-^ mice and IL-36γ^-/-^ mice during *P*. *aeruginos*a infection, as indicated by reduced lung injury scores and lower BAL albumin concentration (Fig 4E). The *P*. *aeruginosa* strain (ATCC 19660) we used in the present study expresses the Type III secretion system (T3SS), which deliver virulence factors to the cytosol of host cells, and the T3SS in *P*. *aeruginosa* promotes lung injury through disruption of alveolar-epithelial barrier [47, 48]. The mechanism (s) by which IL-36 cytokines exacerbate lung injury in *P*. *aeruginosa* pneumonia have not been completely defined. This effect is unlikely to be mediated by PGE2/EP2 signaling, as no differences in BAL albumin levels were noted between EP2 deficient mice or mice treated with NS-398 as compared to control mice post *P*. *aeruginosa* challenge despite differences in lung bacterial burden. Also, we did not find differences in inflammatory leukocyte influx at 6 and 24 h post bacterial administration. We did note reductions in both IL-6 and IL-17, and to a lesser extent TNFα in the BAL fluid of infected IL-36R^-/-^ mice and IL-36γ^-/-^ mice at 24 h post infection, as compared to WT mice. In patients with community-acquired pneumonia, the levels of IL-6 in BAL and plasma are positively correlated with the severity of disease [49, 50]. Moreover, IL-6 deficient mice have been shown to be protected from lung injury and mortality during *P*. *aeruginosa* pulmonary infection [51]. Mechanistically, IL-6 can induces signal transducers and activator of transcription (STAT) 3 activation, and excessive lung STAT3 activation in *P*. *aeruginosa* lung infection has been shown to result in more severe lung injury and increased mortality [52]. IL-36 receptor ligands directly induce the production of IL-6 in PMs, AECs [7] and dendritic cells [31]. These data suggested that IL-6 induced by IL-36γ may be associated with lung injury during *P*. *aeruginosa* infection. We also observed reductions in IL-17 in infected mutant mice, and IL-17 has been shown to mediate injury responses in certain pulmonary infections such as pneumonia caused by influenza virus [53]. However, we did not observe defects in BAL PMN accumulation (Fig 3C) or antimicrobial peptide mRNA expression in IL-36R or IL-36γ deficient mice (S3 Fig), calling into question the physiological significance of reduced IL-17 levels.

In conclusion, this study identifies IL-36γ, released from PMs and AECs and likely other lung cells, as a mediator of impaired lung host immune response and lung injury during *P*. *aeruginosa* infection. Our findings provide fundamental insights into the pathophysiology of *P*. *aeruginosa* induced pneumonia, insights which may have important therapeutic implications.

## Material and methods

### Mice

Specific pathogen-free age- and sex-matched C57BL/6 mice were purchased from The Jackson Laboratory (Bar Harbor, ME, USA). IL-36R^-/-^ mice on a C57BL/6 background were provided by Jennifer Towne from Amgen (Thousand Oaks, CA, USA) [14]. A colony of IL-36α^-/-^ mice bred on a C57BL/6 background was established and provided from RIKEN BRC (Tsukuba, Japan). A colony of IL-36γ^-/-^ mice bred on a C57BL/6 background was established at the University of Michigan (Ann Arbor, MI, USA) [6]. EP-2^-/-^ mice on a C57BL/6 background were kindly given to us by Marc Peters-Golden at the University of Michigan [54]. All mice were housed in specific pathogen-free conditions within the University of Michigan Animal Care Facility.

### *P*. *aeruginosa* and FITC labelling heat-killed *P*. *aeruginosa*

Flagellated *P*. *aeruginosa* strain ATCC 19660 (American Type Culture Collection, Manassas, VA, USA) was used for all of experiments. Bacteria was grown overnight in Difco nutrient broth (BD Biosciences, Franklin Lakes, NJ, USA) at 37°C with constant shaking. Bacteria concentrations were determined by measuring the amount of absorbance at 600nm and compared to a predetermined standard curve based on known colony-forming unit (CFU) values. To prepare heat killed *P*. *aeruginosa*, bacteria was incubated at 65°C for 1h. For FITC labeling, heat-killed *P*. *aeruginosa* was resuspended at 10^9^–10^10^ CFU/mL in 0.1M NaHCO3 (pH 9.0). A total of 0.2 mg/ml FITC (Invitrogen, Carlsbad, CA, USA) in DMSO was added to heat-killed bacteria and incubate in the dark for 1 h on a rocker at room temperature. Following FITC labeling, bacteria was washed three times and responded in 1ml sterile PBS. Aliquots were prepared and store at -80°C.

### Intratracheal infection with *P*. *aeruginosa*

For intratracheally administration, mice were anesthetized by intraperitoneal injection of ketamine and xylazine and then infected 50μl of 2 × 10^5^ CFU by insertion of 24-gauge intravenous catheter into the trachea.

### Bronchoalveolar lavage and sample preparation

BAL was performed as described previously [55]. Mice were euthanized by CO_2_ inhalation. The trachea was exposed and cannulated with 22 G intravenous catheter. BAL was performed with 3 mL PBS containing 5mM EDTA (tree aliquot 1mL of PBS), and then pulmonary circulation was rinsed by 1ml PBS. Lungs were harvested for RNA extraction, immediately snap-frozen in liquid nitrogen. After a collection of leukocytes in BAL fluids, cytospin (113 *g* × 5 min) preparations were made from each sample and stained with modified Wright stain. Differential cell counts of neutrophils and monocytes and macrophages were obtained for at least 400 cells counts in each sample at a magnification of ×1000.

### Quantification of bacterial burden in BAL fluid and spleen

Samples of BAL fluid and homogenized spleen in PBS were serially diluted 10 fold in PBS. 10 μl of each samples were plated on a nutrient agar. Bacterial colonies were counted after the plates were incubated at 37°C for 18 h.

### Histopathological examination

Lungs and trachea were removed from euthanized animals and inflated at 20cm H_2_O with 4% paraformaldehyde through trachea, and fixed for paraffin embedding. All lungs were sectioned and stained with haematoxylin and eosin (H&E). Quantitative analysis of tissue injury was measured using the lung injury scoring system as described [56]. Lung injury scoring system parameters include neutrophils in the alveolar space (A), neutrophils in the interstitial space (B), hyaline membranes (C), proteinaceous debris filling the airspaces (D) and alveolar septal thickening (E). At least 20 random regions were scored 0–2 independently at a magnification of ×400 in a blinded fashion. The final lung injury score per each lungs was calculated as below; score = [(20 × A) + (14 × B) + (7 × C) + (7 × D) + (2 × E)] / (number of fields × 100).

### Murine pulmonary macrophage and alveolar epithelial cell isolation and culture

Murine pulmonary macrophages (PMs) and type II alveolar epithelial cells (AECs) were isolated using the method described previously [57, 58]. Briefly, pulmonary macrophages (consisting of both alveolar and interstitial macrophages) were isolated from dispersed lung digest cells by adherence purification as previously described [57]. For the isolation of murine AECs, the pulmonary vasculature was perfused. The lungs were filled via the trachea with 1.5 ml dispase (Worthington, Lakewood, NJ. USA), then 1.5 ml of low-melting point agarose and finally placed in ice cold PBS. The lungs were submerged in dispase for 45 min at 24°C before the lung tissue was teased from the airways and minced in DMEM with 0.01% DNase. After swirling for 15 min, followed by passage through a series of nylon filters, the cell suspension was collected by centrifugation and incubated with biotinylated Abs (anti-CD32 and anti-CD45; BD Pharmingen, San Diego, CA, USA). After incubation with streptavidin-coated magnetic particles, myeloid cells were removed using a magnetic tube separator. Mesenchymal cells were removed by overnight adherence in a Petri dish and the resulting non-adherent cells were plated on plastic dishes coated with fibronectin. Previous work has shown that the day 3 time point has >90% pure AECs [58]. These cells were treated with live or heat killed *P*. *aeruginosa* at a MOI of 10, LPS (1μg/ml) (Sigma-Aldrich), and recombinant IL-36α (100ng/mL) and IL-36γ (100ng/mL) (R&D Systems Minneapolis, MN, USA).

### *In vitro* phagocytosis assay

The ability of PMs to phagocytosis bacteria was examined using FITC-labeled *P*. *aeruginosa*. PMs isolated from WT mice, IL-36R^-/-^ mice and EP2^-/-^ mice were plated at 1 × 10^6^ cells/well and cultured overnight with or without rIL-36γ (100ng/mL) on 24-well plate. The following day, wells were washed with antibiotics free CM, and PMs were incubated with FITC-labeling or non-labeling heat-killed *P*. *aeruginosa* at a MOI 100. Two hours later, cells were collected by cell scraper and stained with PerCP-Cy5.5-labeling CD45 (BD Pharmigen, San Jose, CA, USA) and PE-labeling F4/80 (BD Pharmigen). Isotype controls were used for all the samples. PMs phagocytosis of FITC-labeled bacteria was analyzed by Attune Acoustic Focusing Cytometer (Thermos Scientific-Applied Biosystems, Foster City, CA, USA).

### *In vitro* bacterial killing assay

Bacterial killing assay was assessed using a modification of protocol previously reported [59]. PMs were seeded on 24-well plate at 1 × 10^6^ cells/well and cultured overnight with or without rIL-36γ (100ng/ml). Following day, PMs were infected with live *P*. *aeruginosa* at a MOI 100. At 30 min after incubation, infected PMs were washed with gentamicin at 100μg/ml solution twice to remove extracellular bacteria, and then cells were lysed to obtain initial CFU, or incubated further at 37°C for more 90 min. Cells were subsequently lysed by 0.1% Triton X-100, followed by serial plating for bacterial CFU quantification.

### Determination of cytokines, albumin and PGE2 production by ELISA

Murine IL-36α and IL-36γ secreted in BAL and CM were measured by previously reported sandwich ELISA method [6]. For human IL-36α and IL-36γ ELISA generation, human recombinant IL-36α and IL-36γ and human anti-IL-36α and anti-IL-36γ polyclonal antibodies were purchased from R&D Systems. Other cytokines/chemokines (TNF-α, IL-6, IL-17 and IL-10; R&D systems) and albumin (Albumin Quantification Kit: Bethyl Laboratories, Montgomery, TX, USA) were quantified using a modified double-ligand method as described. The production of PGE2 were determined using an ELISA Kit according to the manufacture’s protocol (Cayman, Ann Arbor, MI, USA).

### RNA isolation and real-time PCR

RNA was isolated and real time quantitative RT-PCR was performed by AB Step One plus Real-Time PCR System (Thermos Scientific-Applied Biosystems). Predesigned primer and probes of targeted molecules and β-actin as a control were purchased from Integrated DNA Technologies (Coralville, IA, USA). Quantification of β-actin and target genes in each sample set was performed by the standard curve method.

### Western blotting

Cells were digested by RIPA buffer (Sigma-Aldrich) plus protease inhibitors and gels were subjected to electrophoresis as previously described [58]. Membranes were incubated with primary anti-COX-2 antibody (Cayman; diluted 1:100) or β-actin (Sigma-Aldrich; diluted 1:20,000), blots were incubated with a secondary antibody linked to HRP and the signals were developed with an ECL (SuperSignal West Pico Substrate, Pierce Biotechnology, Rockford, IL, USA).

### Study population of patients with *P*. *aeruginosa*-induced ARDS

Patients with ARDS that were enrolled in the Acute Lung Injury Specialized Center of Clinically Oriented Research (SCCOR) randomized trial of granulocyte-macrophages colony stimulating factor administration in ARDS conducted at the University of Michigan between Jan 2004 and October 2007 were studied [60]. We identified patients with *P*. *aeruginosa* induced ARDS who obtained *P*. *aeruginosa* from sterile culture sites including blood or BAL samples, and no other putative pathogens identified. Sixteen patients were matched for these criteria, and 16 plasma and 10 BAL samples were obtained from these patients within seven days from the onset of ARDS. Six plasma and nine BAL samples of healthy subjects were used as control.

### Ethics statement

Animal studies were reviewed and approved by the University Committee on Use and Care of Animals at the University of Michigan in accordance with guidelines of the Care and Use of Laboratory Animals of the National Institutes of Health (protocol #PRO00006295). Experiments using human samples were approved by the University of Michigan Institutional Review Board (IRB#2003–0430 and IRB#2003–0829) and conducted in accordance with the principles expressed in the Declaration of Helsinki. Written informed consent was obtained from participants. If the patients with ARDS received mechanical ventilation under the sedation, and they were not able to make their decision by themselves, we obtained written informed consent from their legal proxy for medical decision making before study inclusion. IRB approved a legally authorized representative to sign proxy informed consent. All patients enrolled in this study were over the age of 18.

### Statistical analysis

Descriptive statics, such as means and standard deviations, were collected. The difference in survival rates was evaluated by the log rank test (Mantel-Cox). Two sets of values were evaluated by the Student’s t-test, and more than three sets of value were evaluated by ANOVA, followed by the Turkey’s multiple comparison test. Data analysis was conducted using Graphpad prism 6 (GraphPad Software, La Jolla, CA, USA). A *P* value of <0.05 was considered satirically significance.

## Supporting information

S1 Fig Caspase-1 does not contribute to the induction and secretion of IL-36 cytokines by pulmonary macrophages during *P*. *aeruginosa* infection. (A, B) Primary PMs isolated from WT mice were incubated with LPS (1μg/ml), live and heat-killed (HK) *P*. *aeruginosa* at a MOI 10. (A) The cell lysate from LPS, live and heat killed bacteria treated PMs were subjected to western blot with caspase-1 p10 subunit specific antibody (1:500). Precursor caspase-1, caspase-1 p10 and β-actin was shown. (B) The production of IL-1β was examined by ELISA. (C-E) Primary PMs isolated WT mice were pre-incubated with or without caspase-1 inhibitor (20μM) for 1h, and then stimulated with *P*. *aeruginosa* at a MOI 10. In some experiments, cells were stimulated with or without ATP treatment (50nM) for 20 min after 24 h stimulation, and then culture medium (CM) were collected. (C) The production of IL-1β in CM without ATP treatment was measured by ELISA. (D) mRNA of IL-36α and IL-36γ were analyzed by real-time PCR. (E) The production of IL-36α and IL-36γ in CM, which were treated with or without ATP, were measured by ELISA. * *p*<0.05, # *p*<0.01, § *p*<0.001, ¶ *p*<0.0001, n.s. not significant, compared with medium only or as indicated.
(TIF)

S2 Fig *P*. *aeruginosa* does not activate caspase-3/7 in PMs and AECs. Primary PMs and AECs isolated from WT mice were incubated with live and heat killed (HK) P. aeruginosa at MOI 10. Activity of Caspase-3/7 in PMs (left panel) and AECs (right panel) was measured using Apo-ONE Homogeneous Caspase-3/7 Assay (Promega, Madison, WI, USA). Data are shown as means ± SEM.
(TIF)

S3 Fig Expression of antimicrobial peptide during *P*. *aeruginosa* infection. WT, IL-36 receptor deficient (IL-36R^-/-^) and IL-36γ deficient (IL-36γ^-/-^) mice were intratracheally infected with 2.0 × 10^5^ CFU P. aeruginosa. Transcript products of β-defensin 3 (left panel) and cathelicidin antimicrobial peptide (CAMP) (right panel) in the lungs of untreated, 6 h and 24 h after *P*. *aeruginosa* infection. mRNA was analyzed by real-time PCR. All data are shown as means ± SD of 4–5 mice/group.
(TIF)

S4 Fig IL-36 agonists did not changes the production of PGE2. Primary AECs isolated from WT and treated with recombinant IL-36α (100ng/ml) and IL-36γ (100ng/ml) for 24 h. (A) The expression of prostaglandin-endoperoxide synthase 2/cyclooxygenase 2 (Ptgs2/COX-2) mRNA in PMs was analyzed by real-time PCR. (B) The protein production of PGE2 in CM by PMs was examined by ELISA. Data are shown as means ± SEM.
(TIF)

S5 Fig The phagocytic ability and bacterial killing of bone-marrow derived neutrophils and pulmonary macrophages in response to *P*. *aeruginosa*. (A,C) Bone-marrow derived neutrophils (BMDNs) were harvested from mouse femur/tibia using density gradient centrifugation method. BMDNs or primary pulmonary macrophages (PMs) isolated from WT mice, IL-36R^-/-^ mice and IL-36γ^-/-^ were plated at 5 × 10^5^ or 1 × 10^6^ cells/well, respectively. BMDNs were incubated for one hour and PMs were incubated for 18 h. After incubation, cells were washed with antibiotics free culture medium and incubated with FITC-labeled or non-labeled heat-killed *P*. *aeruginosa* at a MOI 300 for BMDNs and at a MOI 100 for PMs. After 2h incubation, cells were collected and analyzed the phagocytic response as FITC positive cells by flow cytometry. (B, D, E) BMDMs (B) and PMs (D) were seeded at 1 × 10^6^ cells/well. After incubation, BMDMs for one hour and PMs for 18 h. (E) COX-2 inhibitor (NS-398) or vehicle were treated in with or without rIL-36γ treated PMs (1 × 10^6^ cells/well) for 18h cells. After incubation, cells were washed with antibiotics free culture medium and incubated with live *P*. *aeruginosa* at a MOI 100, respectively. PMs were harvested at 30 min to quantify CFU as the initial time point, or incubated further for an additional 90 min. CFU/10^6^ PMs were obtained each samples by subsequent dilution method. After 30 min, cells were washed with gentamycin solution (100μg/mL) twice PMs to obtain initial colony forming unit (CFU) or incubated further for an additional 90 min. CFU/10^6^ BMDMs or PMs were obtained each samples by subsequent dilution method. Data (means ± SEM) are representative of two independent experiments. # *p*<0.01, compared as indicated.
(TIFF)

S6 Fig Role of IL-10 in *P*. *aeruginosa* infection *in vivo*. WT mice were administrated anti-IL-10 antibody or rat IgG1 1 h before 2.0 × 10^5^ CFU *P*. *aeruginosa* challenge. Bacterial counts in BAL (left panel) and homogenized spleen samples (right panel) were examined. Each group consisted of 4 mice. Data are shown as mean.
(TIFF)
