# Supplementary figures and images for: Interleukin-36γ and IL-36 receptor signaling mediate impaired host immunity and lung injury in cytotoxic Pseudomonas aeruginosa pulmonary infection: Role of prostaglandin E2
Source: PLoS Pathog. 2017 Nov 22;13(11):e1006737. doi: 10.1371/journal.ppat.1006737 (PMC5718565; doi:10.1371/journal.ppat.1006737)

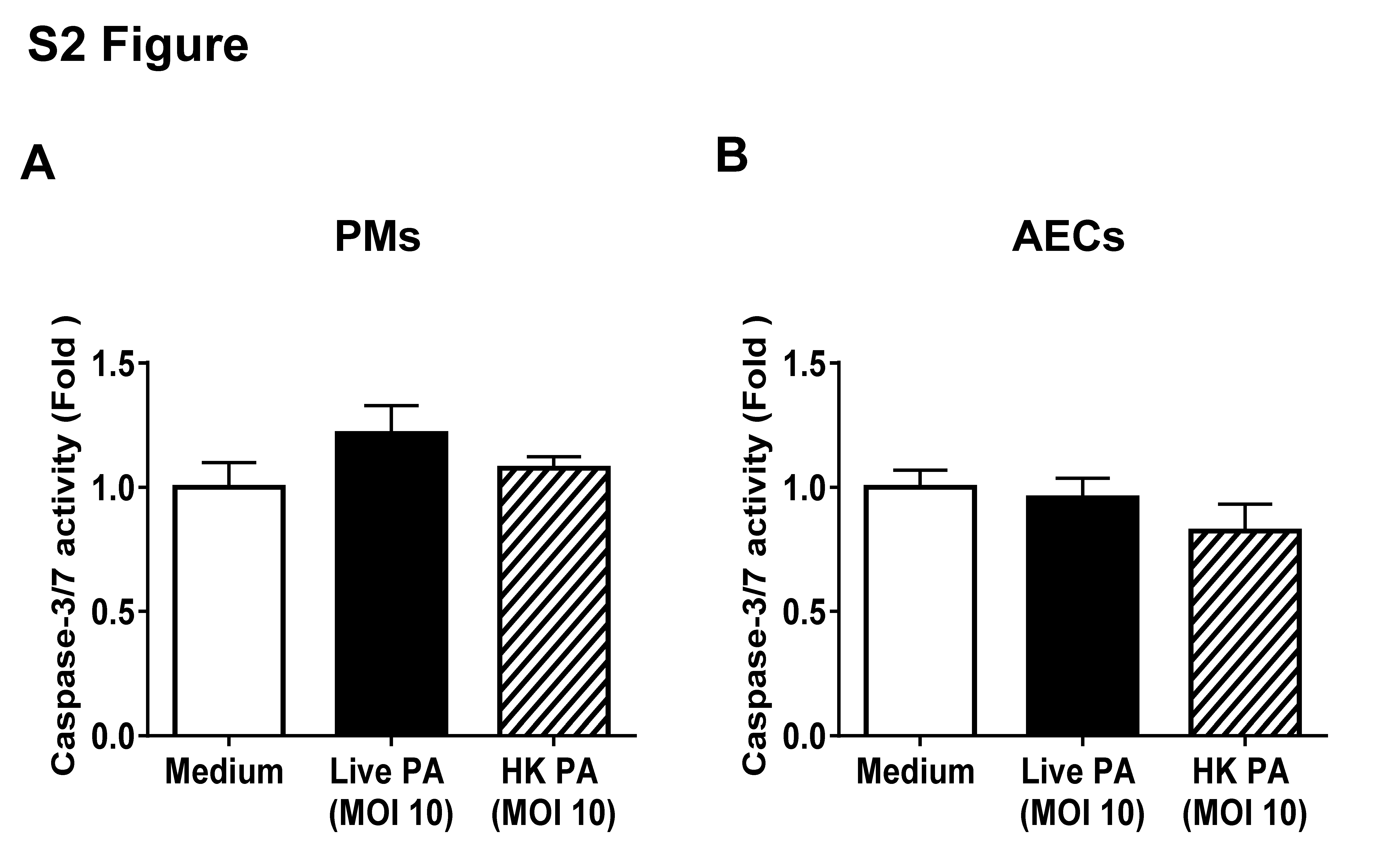

Supplement: S2 Fig — Primary PMs and AECs isolated from WT mice were incubated with live and heat killed (HK) P. aeruginosa at MOI 10. Activity of Caspase-3/7 in PMs (left panel) and AECs (right panel) was measured using Apo-ONE Homogeneous Caspase-3/7 Assay (Promega, Madison, WI, USA). Data are shown as means ± SEM. (TIF) [file ppat.1006737.s002.tif]

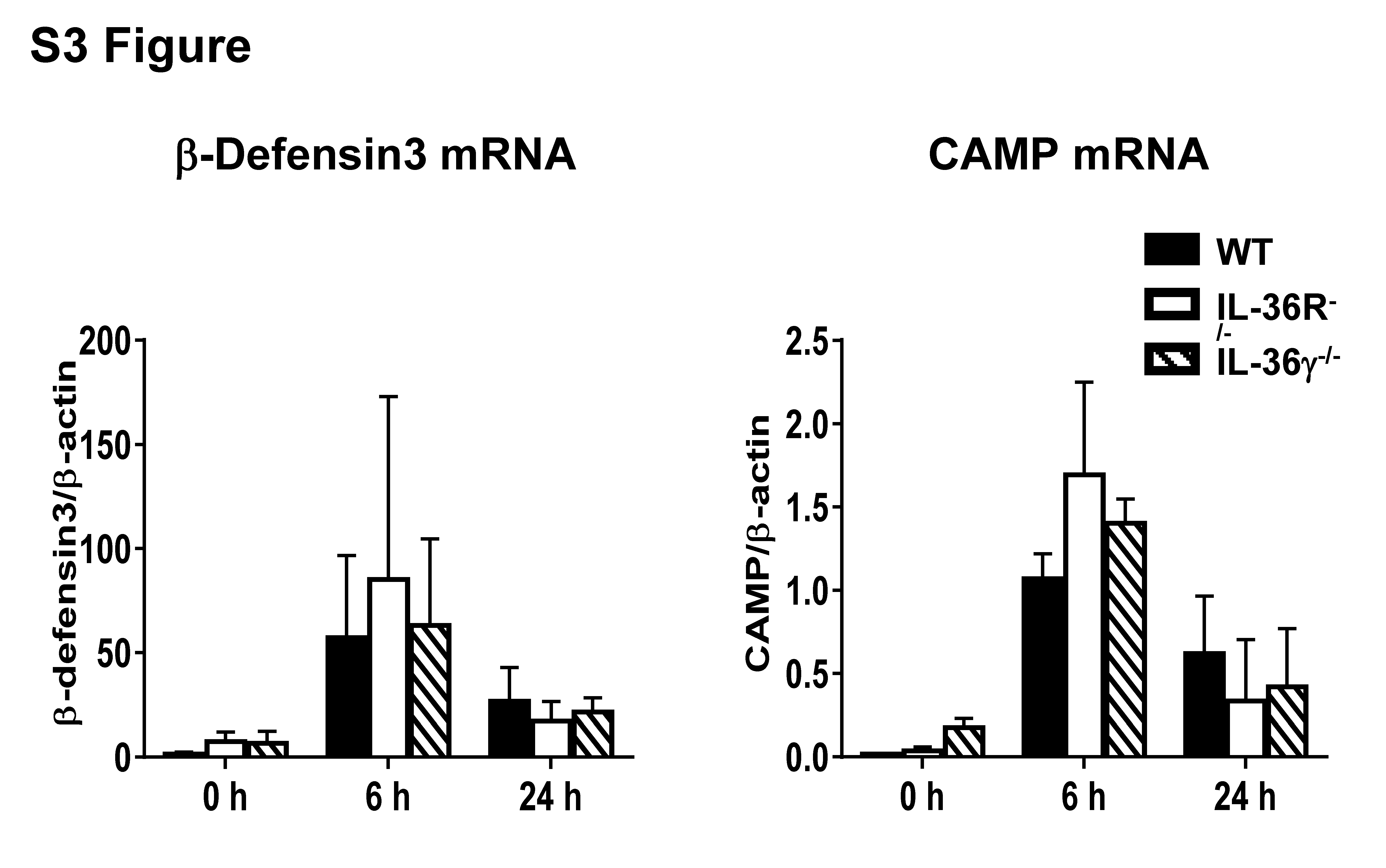

Supplement: S3 Fig — WT, IL-36 receptor deficient (IL-36R-/-) and IL-36γ deficient (IL-36γ-/-) mice were intratracheally infected with 2.0 × 10^5^ CFU P. aeruginosa. Transcript products of β-defensin 3 (left panel) and cathelicidin antimicrobial peptide (CAMP) (right panel) in the lungs of untreated, 6 h and 24 h after P. aeruginosa infection. mRNA was analyzed by real-time PCR. All data are shown as means ± SD of 4–5 mice/group. (TIF) [file ppat.1006737.s003.tif]

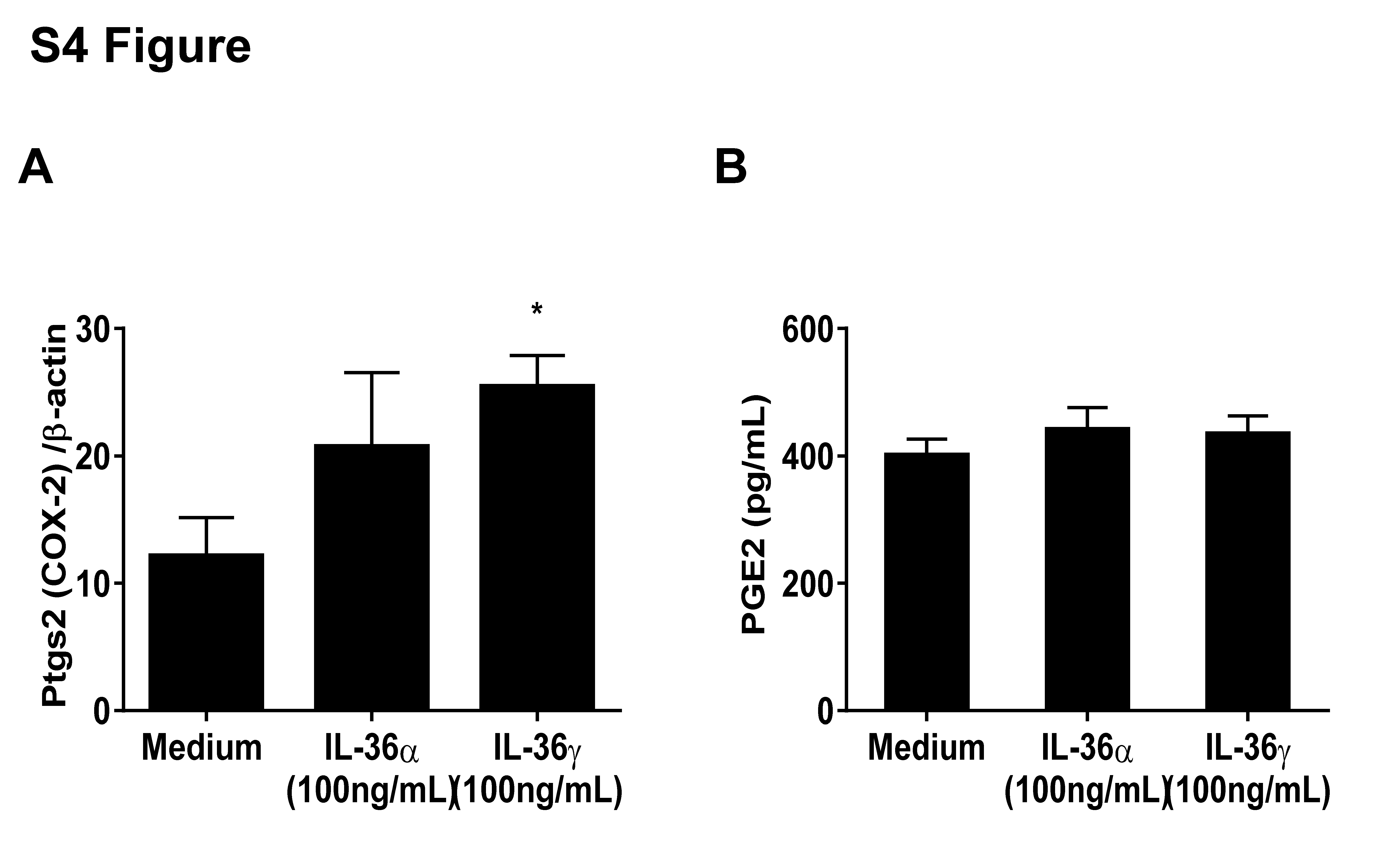

Supplement: S4 Fig — Primary AECs isolated from WT and treated with recombinant IL-36α (100ng/ml) and IL-36γ (100ng/ml) for 24 h. (A) The expression of prostaglandin-endoperoxide synthase 2/cyclooxygenase 2 (Ptgs2/COX-2) mRNA in PMs was analyzed by real-time PCR. (B) The protein production of PGE2 in CM by PMs was examined by ELISA. Data are shown as means ± SEM. (TIF) [file ppat.1006737.s004.tif]

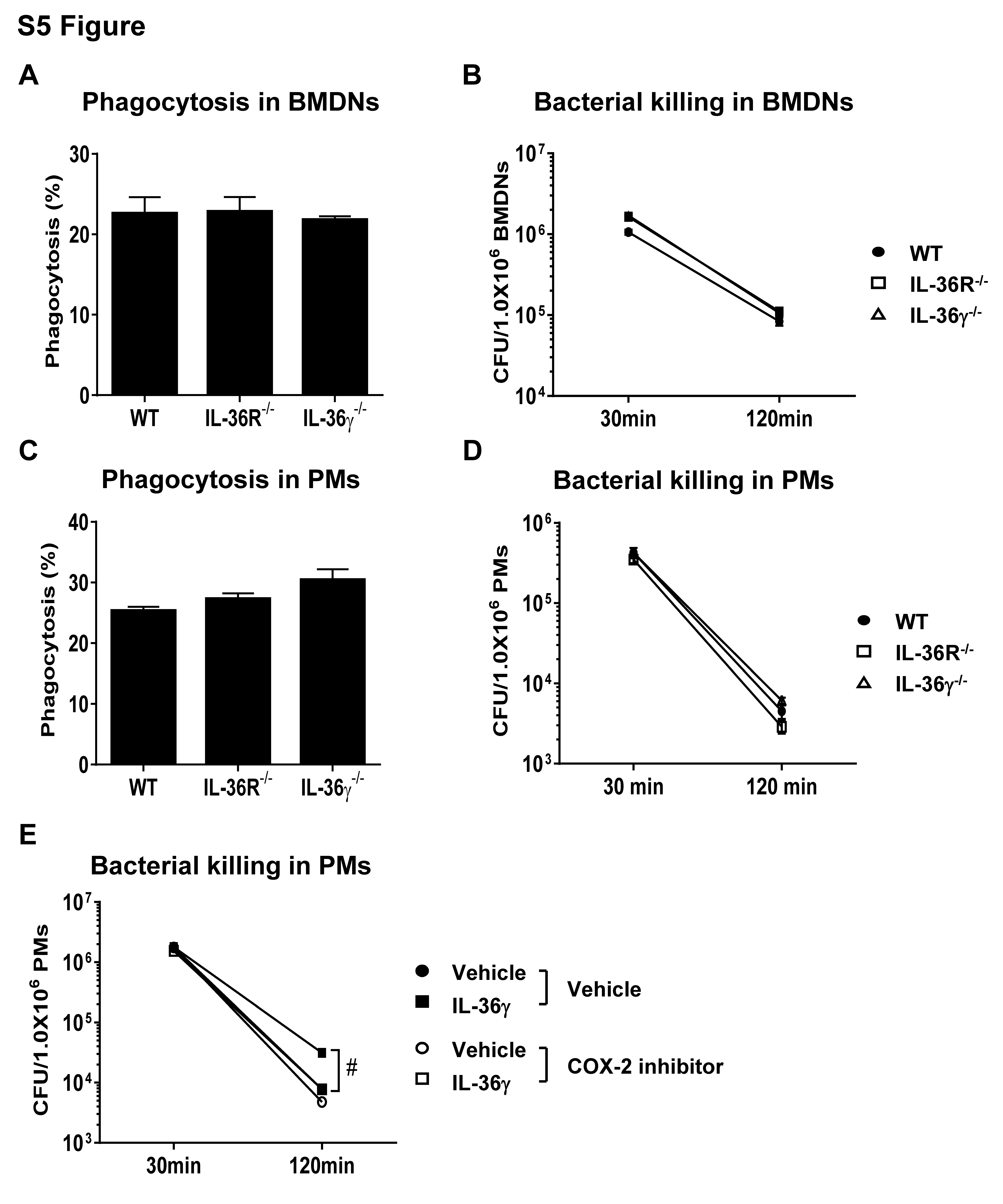

Supplement: S5 Fig — (A,C) Bone-marrow derived neutrophils (BMDNs) were harvested from mouse femur/tibia using density gradient centrifugation method. BMDNs or primary pulmonary macrophages (PMs) isolated from WT mice, IL-36R-/- mice and IL-36γ-/- were plated at 5 × 10^5^ or 1 × 10^6^ cells/well, respectively. BMDNs were incubated for one hour and PMs were incubated for 18 h. After incubation, cells were washed with antibiotics free culture medium and incubated with FITC-labeled or non-labeled heat-killed P. aeruginosa at a MOI 300 for BMDNs and at a MOI 100 for PMs. After 2h incubation, cells were collected and analyzed the phagocytic response as FITC positive cells by flow cytometry. (B, D, E) BMDMs (B) and PMs (D) were seeded at 1 × 10^6^ cells/well. After incubation, BMDMs for one hour and PMs for 18 h. (E) COX-2 inhibitor (NS-398) or vehicle were treated in with or without rIL-36γ treated PMs (1 × 10^6^ cells/well) for 18h cells. After incubation, cells were washed with antibiotics free culture medium and incubated with live P. aeruginosa at a MOI 100, respectively. PMs were harvested at 30 min to quantify CFU as the initial time point, or incubated further for an additional 90 min. CFU/106 PMs were obtained each samples by subsequent dilution method. After 30 min, cells were washed with gentamycin solution (100μg/mL) twice PMs to obtain initial colony forming unit (CFU) or incubated further for an additional 90 min. CFU/106 BMDMs or PMs were obtained each samples by subsequent dilution method. Data (means ± SEM) are representative of two independent experiments. # p<0.01, compared as indicated. (TIFF) [file ppat.1006737.s005.tiff]

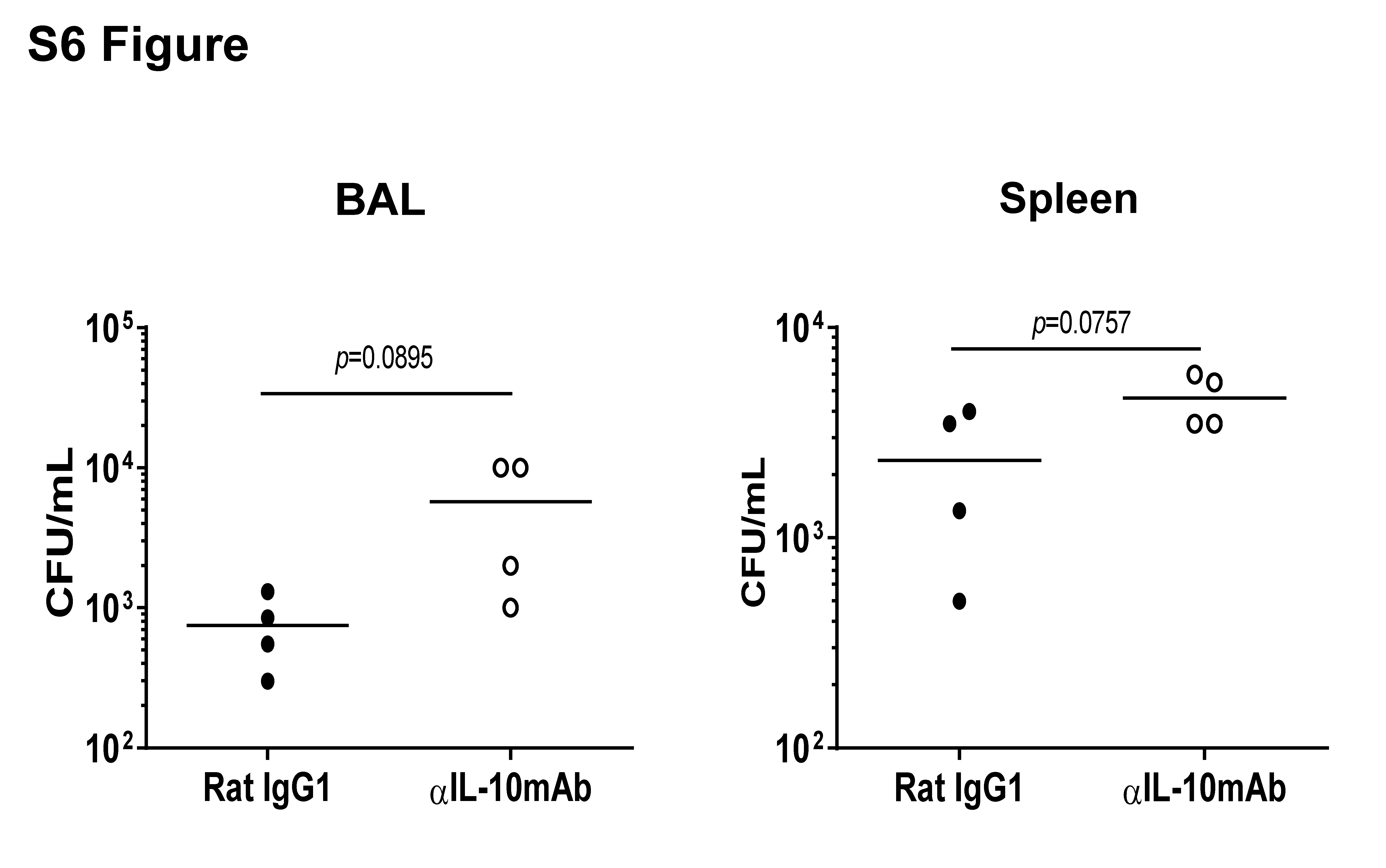

Supplement: S6 Fig — WT mice were administrated anti-IL-10 antibody or rat IgG1 1 h before 2.0 × 10^5^ CFU P. aeruginosa challenge. Bacterial counts in BAL (left panel) and homogenized spleen samples (right panel) were examined. Each group consisted of 4 mice. Data are shown as mean. (TIFF) [file ppat.1006737.s006.tiff]
